# Agreeing on global research priorities for medication safety: an international prioritisation exercise

**DOI:** 10.7189/jogh.09.010422

**Published:** 2019-06

**Authors:** Aziz Sheikh, Igor Rudan, Kathrin Cresswell, Neelam Dhingra-Kumar, Mei Lee Tan, Minna L Häkkinen, Liam Donaldson, on behalf of the World Health Organization's Management Team on Research Priorities for Medication Safety

**Affiliations:** 1 Usher Institute of Population Health Sciences and Informatics, University of Edinburgh, Edinburgh, UK; 2 World Health Organization, Geneva, Switzerland; 3 London School of Hygiene and Tropical Medicine, London, UK

## Abstract

**Objectives:**

Medication errors continue to contribute substantially to global morbidity and mortality. In the context of the recent launch of the World Health Organization’s (WHO) Third Global Patient Safety Challenge: *Medication Without Harm*, we sought to establish agreement on research priorities for medication safety.

**Methods:**

We undertook a consensus prioritisation exercise using an approach developed by the Child Health and Nutrition Research Initiative. Based on a combination of productivity and citations, we identified leading researchers in patient and medication safety and invited them to participate. We also extended the invitation to a further pool of experts from the WHO Global Patient Safety Network. All experts independently generated research ideas, which they then independently scored based on the criteria of: answerability, effectiveness, innovativeness, implementation, burden reduction and equity. An overall Research Priority Score and Average Expert Agreement were calculated for each research question.

**Findings:**

131 experts submitted 333 research ideas, and 42 experts then scored the proposed research questions. The top prioritised research areas were: (1) deploying and scaling technology to enhance medication safety; (2) developing guidelines and standard operating procedures for high-risk patients, medications and contexts; (3) score-based approaches to predicting high-risk patients and situations; (4) interventions to increase patient medication literacy; (5) focused training courses for health professionals; and (6) universally applicable pictograms to avoid medication-related harm. Whilst there was a focus on promoting patient education and involvement across resource settings, priorities identified in high-resource settings centred on the optimisation of existing systems through technology. In low- and middle-resource settings, priorities focused on identifying systemic issues contributing to high-risk situations.

**Conclusions:**

WHO now plans to work with global, regional and national research funding agencies to catalyse the investment needed to enable teams to pursue these research priorities in medication safety across high-, middle- and low-resource country settings.

Medication errors are common and are responsible for considerable – potentially avoidable – morbidity and mortality [1]. They are also costly for patients, health systems and society; globally medication errors impose an estimated financial burden of US $42 billion per year, accounting for almost 1% of total expenditure on health worldwide [2].

Most studies of the frequency and nature of medication error have come from high-resource country settings. These have found that around 2-3% of clinical encounters in primary care settings and 10% involving hospital in-patients will result in errors [3-10]. Not all will be clinically important, but medication errors have been found to be the commonest resulting in harm [11]. Research in low-resource health care settings suggests a broadly comparable frequency of medication-related harm [6-12]. This body of work is challenging to interpret, because of the heterogeneity in the definitions used, patient populations studied and methodologies employed. Despite these challenges, the over-riding message from this body of evidence is very clear: medication errors affect patients of all ages, both sexes, occur globally across all health care settings, and, most importantly, they are largely preventable.

The World Health Organization (WHO) established two previous Global Patient Safety Challenges. Each Challenge sought to highlight a major patient safety problem impacting all countries and health systems. The first of these, Clean Care is Safer Care (2005) focused on reducing health care-associated infections and the second [13], Safe Surgery Saves Lives (2007), aimed to improve the safety of surgical processes [14]. Building on the success of these global initiatives, WHO launched the third WHO Global Patient Safety Challenge: *Medication Without Harm* in 2017 [15]. This seeks to facilitate a range of strategic initiatives with the aim of improving medication safety globally. Research is fundamental to these global, regional and national efforts. To inform these deliberations, we sought to define research priorities for medication safety using an inclusive, systematic and replicable process.

## Methods

### Origins

We established a management team to identify global research priorities for medication safety. After reviewing available approaches for research prioritisation, we decided to employ the Child Health and Nutrition Research Initiative (CHNRI) method [16]. This research priority setting technique was introduced in 2007 and has now successfully been applied in over 100 different exercises, a number of which have also been led by WHO [17] (see **Box 1**). Revised guidelines for application of the method, based on the experience of its use, have recently been published [18-23]. This followed extensive deliberation on what was learnt from working with funders, researchers and other stakeholders over the last decade, as well as the development of a revised conceptual framework and validation of the key concepts that CHNRI relies on. In June 2017, we developed a protocol to guide the process for setting priorities in medication and patient safety research globally. A small management team (including the authors of this report) coordinated the steps of the WHO priority setting exercise.

BOX 1 **The CHNRI method for setting research priorities**

The CHNRI method uses the principle of crowdsourcing to score ideas against a pre-defined set of criteria. This enables funders and policymakers to view the strengths, the weaknesses, and relative ranking of each proposed research idea, based on submitted opinions of a larger number of experts. This method uses a systematic, transparent, and democratic approach to priority setting. While it allows researchers to independently generate and score research questions, it also involves funders, policymakers, and other stakeholders at an early stage of the process, ensuring their ownership of the outcomes. The CHNRI method has thus far been implemented in about 100 studies led by multilateral organisations (eg, WHO, United Nations International Children's Emergency Fund (UNICEF)), national governments (eg, India, South Africa), and funders (eg, The Bill and Melinda Gates Foundation) to set research priorities in areas ranging from the reduction of global child mortality, dementia, or disability to the efficient execution of national health plans (eg, in China). The recognised advantages of this method include its systematic nature, transparency and replicability, clearly defined context and criteria, involvement of the funders, stakeholders and policy makers, a structured way of obtaining information, informative and intuitive quantitative outputs, studying the level of agreement over each proposed research idea, and independent scoring of many experts, thus limiting the influence of individuals on the rest of the group [18-24].

### Expert input

We identified 598 experts in medication safety from across the world. We did so by searching the Web of Science's Core Collection for the most productive authors in the preceding five-year period, or those who were lead authors of the top 1% most cited research articles. The key words used to identify the experts were “medication safety” and “patient safety”. After removing duplicate names, we invited the resulting 457 researchers to participate. Each expert was invited to generate up to three research ideas and then systematically rank these using pre-agreed criteria (detailed below). We in addition approached 190 persons who expressed interest in medication safety through the WHO’s Global Patient Safety Network. A total of 131 invitees agreed to participate and submitted their research ideas.

The management team then scrutinised the submitted ideas and ensured that the wording of each idea fitted the format for the scoring process. This led to a consolidated list of 333 unique research ideas, which were then thematically organised into 33 broader categories, each containing between three and 30 research ideas.

The larger participating scorer group, comprising 42 experts, scrutinised the list of questions and agreed on the context and the criteria for scoring. The context was defined as “global.” This meant that some proposed research ideas were scored differently because they were not feasible in specific settings. We asked the scorers to state whether the context they considered for the scoring process was for high-resource or low-resource settings. The resulting data were used for sub-analyses (see later).

### Criteria

The timeframe within which the results were expected from proposed research was 5-10 years. Six independent criteria were agreed and used to discriminate between the many proposed research questions identified:

- *Answerability*: Is this research question likely to be answered using the proposed methods and approaches?
- *Effectiveness*: Is this research question likely to lead to interventions that will effectively reduce the burden of medication-related harm?
- *Innovativeness*: Is this research question truly novel, making good use of overall technological and scientific progress?
- *Implementation*: Is this research question likely to lead to interventions or solutions that could be readily implemented?
- *Burden reduction*: Is this research question likely to lead to a significant reduction in medication-related harm?
- *Equity*: Is this research question likely to reduce inequity in the population?

All invited contributors were asked to score each submitted research question using these pre-defined criteria.

### Scoring

Experts were offered four response options for scoring: 0 (unlikely to meet the criterion); 0.5 (not sure if it can meet the criterion); 1 (likely to meet the criterion); or left blank if the expert felt insufficiently informed to make a judgment. The scores for each criterion ranged from 0-100%, and the overall research priority score (RPS) assigned to each research question was a simple mean of all six criteria-specific scores. Average expert agreement (AEA), defined as the level of agreement among scorers, was also calculated for each research question, as the frequency of the mode (ie, the most common score divided by the total number of scores).

## RESULTS

We received scores for the 333 proposed research ideas from 42 experts (see Table S1 in **Online Supplementary Document**). Most experts (n = 27) scored with a high-resource setting in mind. Five experts did not indicate which setting they had in mind while scoring. We present the scores from the overall pool of 42 experts in **Table 1** and Table S2 in **Online Supplementary Document**, and the results by resource context in **Table 2** and Table S3 in **Online Supplementary Document** (high-resource context), and in **Table 3** and Table S4 in **Online Supplementary Document** (low-resource context). **Table 4** shows the 10 research questions with the most divergent scores based on the measure of AEA, which has a maximum theoretical range of 25%-100%. **Table 5** shows the lowest ranked research priorities.

**Table 1:** The top 20 research priorities among the 333 proposed research questions based on the scores from 42 experts in medication safety*

RANK ALL	RESEARCH QUESTION	ANSWERABLE	EFFECTIVE	INNOVATIVE	IMPLEMENTABLE	BURDEN REDUCED	EQUITABLE	RPS	AEA
**1**	To assess how the incidence of harm due to prescribing errors can be reduced by different interventions in low- and middle-income countries.	94	95	74	84	97	91	89.2	0.643
**2**	To assess the prevalence, main factors responsible and the effective interventions for preventing severe avoidable medication-related patient harm in resource-limited settings through pilot studies.	90	92	76	85	90	88	86.8	0.575
**3**	To identify affordable and effective methods of improving medication literacy among patients in resource limited settings	91	91	73	89	87	89	86.7	0.615
**4**	To develop a predictive algorithm to identify individuals who are at risk of serious medication-related harm.	88	91	90	79	94	76	86.2	0.742
**5**	To investigate the role of health communication strategies to support patients with limited language proficiency, health literacy and education in taking medications safely.	89	88	73	85	85	95	85.8	0.571
**6**	To assess the impact of increasing the amount of trained human resources to reduce medication errors in low- and middle-income countries	91	87	79	80	90	81	84.6	0.599
**7**	To develop and validate a complexity score (c-score) to identify the patients who are at risk of readmission in 30 d due to medication errors which could be used by pharmacists and physicians	91	85	79	90	88	72	84.3	0.631
**8**	To improve medication safety for in-patients, through the application of ergonomics and human factors in the organization of the medications flow: order, distribution, stocking, preparation and administration.	92	86	76	86	89	75	83.9	0.575
**9**	To identify the most effective empowerment methods and tools for patients and their caregivers to speak up when they see the potential for medication-related harm, especially applicable to patients in LMICs, as often the most impacted individuals are poorer and less educated.	85	79	84	79	82	94	83.6	0.595
**10**	To develop and validate a complexity score (c-score) for patients in need for de-prescribing which would help the physicians or pharmacists identify the high-risk patients who might develop drug-drug interactions.	91	84	80	86	84	75	83.3	0.563
**11**	To identify and develop globally applicable pictograms for selected high-risk medications which would convey the critically important safety information	87	82	72	83	88	87	83.1	0.536
**12**	To conduct a study investigating the types of medication-related harm that occur in transitions between hospitals and primary care settings in LMIC.	97	86	63	83	89	80	82.8	0.571
**13**	To create patient knowledge-building tools for medication safety with critical thinking to ensure they are usable for people with low level of literacy, in a reliable format and addressing the role of internet as an information source.	88	83	80	81	78	86	82.6	0.623
**14**	To investigate how technologies could be appropriately implemented and scaled in LMICs to better ensure that drugs are not spoiled, diverted, counterfeited, and that supply chain performance is optimized to avoid stock outs and drug shortages.	83	90	70	83	84	82	82.0	0.452
**15**	To compare the benefits of pictorial information in medication instructions to written instructions alone, in improving medication safety. To what extent, in what contexts and formats is pictorial information most beneficial?	88	78	80	80	76	83	81.0	0.500
**16**	To identify what national strategies and/or policies for medication safety across high-, middle-, and low-income countries exist. What gaps remain in identifying and implementing these prevention strategies/policies?	95	84	63	75	82	85	80.8	0.540
**17**	To evaluate the impact of medication reconciliation in preventing medication errors in low-income countries.	94	85	63	73	85	83	80.6	0.563
**18**	To identify indicators of medication safety that have been utilised in low-resource settings. What is known about their validity, reliability, and feasibility, and what potential indicators should be introduced?	93	80	69	83	78	80	80.6	0.508
**19**	To investigate how to ensure patient safety for patients utilizing oral home-based chemotherapy administration: maximising patient education and monitoring systems.	91	85	76	86	77	69	80.6	0.548
**20**	To identify the reliable easily measured indicators to assess medication safety both at a facility level and at national level	88	83	67	86	80	73	79.6	0.540

RPS – research priority score, AEA – average expert agreement

*Specific scores, ranging from 0-100, are presented for each of the 6 priority-setting criteria: answerability, effectiveness, innovativeness, implementability, potential for burden reduction and equitability. Questions are ranked according to their overall research priority scores (RPS), which also has a maximum theoretical range of 0%-100%. Average expert agreement, which can theoretically range from 25%-100%, is also provided for each question.

**Table 2:** Top 10 research priorities among the 333 proposed research questions based on the scores from 27 experts in medication safety who were scoring mainly with a high-resource context in mind, and who represent a subset of the 42 scorers*

RANK HIC	RESEARCH QUESTION	ANSWERABLE	EFFECTIVE	INNOVATIVE	IMPLEMENTABLE	BURDEN REDUCED	EQUITABLE	RPS	AEA
1	To compare the benefits of pictorial information in medication instructions to written instructions alone, in improving medication safety. To what extent, in what contexts and formats is pictorial information most beneficial?	97	84	91	87	81	95	89.1	0.580
2	To identify and develop globally applicable pictograms for selected high-risk medications which would convey the critically important safety information	93	88	80	89	94	90	89.0	0.599
3	To investigate how technologies could be appropriately implemented and scaled in LMICs to better ensure that drugs are not spoiled, diverted, counterfeited, and that supply chain performance is optimized to avoid stock outs and drug shortages.	91	100	77	88	90	87	88.7	0.500
4	To assess how the incidence of harm due to prescribing errors can be reduced by different interventions in low- and middle-income countries.	95	97	71	75	100	87	87.4	0.568
5	To investigate the role of health communication strategies to support patients with limited language proficiency, health literacy and education in taking medications safely.	90	86	71	85	83	100	85.9	0.599
6	To develop a predictive algorithm to identify individuals who are at risk of serious medication-related harm.	82	92	86	74	96	78	84.7	0.722
7	To identify affordable and effective methods of improving medication literacy among patients in resource limited settings	89	84	79	87	83	84	84.6	0.549
8	To identify and create recommendations for the most effective approach to decision support alerts in electronic prescribing systems, the optimum sensitivity and specificity and criteria which should be used to enable prescriber to receive alerts but not receive alert fatigue.	96	89	74	91	89	68	84.6	0.667
9	To create patient knowledge-building tools for medication safety with critical thinking to ensure they are usable for people with low level of literacy, in a reliable format and addressing the role of internet as an information source.	89	82	77	80	79	96	83.7	0.617
10	To develop and validate a complexity score (c-score) for patients in need for de-prescribing which would help the physicians or pharmacists identify the high-risk patients who might develop drug-drug interactions.	93	81	76	88	86	79	83.7	0.580

RPS – research priority score, AEA – average expert agreement

*Specific scores, ranging from 0-100, are presented for each of the 6 priority-setting criteria: answerability, effectiveness, innovativeness, implementability, potential for burden reduction and equitability. Questions are ranked according to their overall research priority scores (RPS), which also has a maximum theoretical range of 0%-100%. Average expert agreement, which can theoretically range from 25%-100%, is also provided for each question.

**Table 3:** Top 10 research priorities among the 333 proposed research questions based on the scores from 10 experts in medication safety who were scoring mainly with a low-resource context in mind, and who represent a subset of the 42 scorers*

RANK LMICs	RESEARCH QUESTION	ANSWERABLE	EFFECTIVE	INNOVATIVE	IMPLEMENTABLE	BURDEN REDUCED	EQUITABLE	RPS	AEA
1	To assess and identify the weak links in the medication safety process chain to consolidate the local systems and resolve the occurring difficulties and differences in practice.	88	100	94	94	94	100	94.8	0.733
2	To assess the prevalence, main factors responsible and the effective interventions for preventing severe avoidable medication-related patient harm in resource-limited settings through pilot studies.	94	94	100	89	94	94	94.3	0.750
3	To investigate the impact of addressing high alert medications on morbidity and mortality in two pilot sites, one in LMIC and one HIC.	100	100	88	94	89	89	93.3	0.800
4	To identify what national strategies and/or policies for medication safety across high-, middle-, and low-income countries exist. What gaps remain in identifying and implementing these prevention strategies/policies?	94	100	75	94	100	94	92.7	0.717
5	To identify the most effective empowerment methods and tools for patients and their caregivers to speak up when they see the potential for medication-related harm, especially applicable to patients in LMICs, as often the most impacted individuals are poorer and less educated.	89	90	90	95	94	95	92.2	0.833
6	What are the most frequent causes of severe, avoidable medication-related harm in high-, middle-, and low-income countries? If this is not known, what steps need to be taken to build and/or strengthen surveillance systems to identify medication-related harm?	94	100	75	94	94	94	92.0	0.750
7	To identify and create new indicators and metrics for medication safety to measure better the impact of medication safety work.	93	93	93	93	93	86	91.7	0.583
8	To assess the reporting and learning of medication error systems at global and regional level and their impact on system change	94	89	81	94	89	100	91.2	0.750
9	To evaluate the prevalence of unnecessary medications and food supplements, drug-drug interactions and drug-disease interactions among patients who take multiple medications.	94	100	69	94	94	94	91.1	0.767
10	To investigate the correlations between patient education and engagement with adherence to medication, inappropriate prescriptions and adverse drug events; and to identify which education tools are effective and sustainable.	94	95	80	100	95	80	90.7	0.850

RPS – research priority score, AEA – average expert agreement

*Specific scores, ranging from 0-100, are presented for each of the 6 priority-setting criteria: answerability, effectiveness, innovativeness, implementability, potential for burden reduction and equitability. Questions are ranked according to their overall research priority scores (RPS), which also has a maximum theoretical range of 0%-100%. Average expert agreement, which can theoretically range from 25%-100%, is also provided for each question.

**Table 4:** The 10 most controversial research questions among the 333 proposed research questions based on the measure of average expert agreement, which has a maximum theoretical range of 25%-100%*

RANK ALL	RESEARCH QUESTION	ANSWERABLE	EFFECTIVE	INNOVATIVE	IMPLEMENTABLE	BURDEN REDUCED	EQUITABLE	RPS	AEA
**321**	To compare generic marking of every individual medication and dosage against existing medication in improving medication safety?	60	48	45	45	40	43	46.9	0.230
**287**	To conduct research into the development of expert systems encompassing a wide scope of patient information (including age, gender, genetic makeup, laboratory tests), to aid as a clinical decision support.	54	56	54	54	61	54	55.2	0.278
**314**	To perform an observational study to identify which laboratory tests can early diagnose a medication error.	48	46	58	44	46	54	49.5	0.282
**242**	To develop and validate models focused on aspects of hospital layout and health care worker/patient flow to reduce HAIs	73	54	55	66	64	52	60.6	0.282
**317**	To evaluate the efficacy of generic antibiotics compared to their original patented brand. Do they have the same impact on antibiotic-resistant bacteria in the digestive flora?	64	44	44	46	46	46	48.5	0.282
**300**	To determine factors that drive spread of HAIs and investigate new approaches that minimize the role of the health care environment in the spread of germs	68	58	39	56	50	44	52.5	0.282
**331**	To conduct an exploratory study on the conditions and regulations needed to adopt the prescription to OTC switch.	52	32	38	43	27	43	39.4	0.282
**322**	To conduct a study exploring implementation methods of drug classification systems in LMIC.	58	40	39	52	42	46	46.1	0.290
**319**	To assess the consequences to the individual's well-being and to their effectiveness when the workplace pursues complete elimination of avoidable harm.	50	50	52	46	48	39	47.6	0.290
**210**	To conduct a study investigating the impact of procurement based on clinical efficacy and safety, with the use of longitudinal data analytics thereby optimising benefits and minimising harm.	66	69	64	62	65	54	63.2	0.294

RPS – research priority score, AEA – average expert agreement

*The scores from 42 experts in medication safety contributed to this ranking. Overall ranks and scores (RPS) are also provided for each question.

**Table 5:** The 20 lowest-ranked research questions among the 333 proposed research questions based on the scores from 42 experts in medication safety*

RANK ALL	RESEARCH QUESTION	ANSWERABLE	EFFECTIVE	INNOVATIVE	IMPLEMENTABLE	BURDEN REDUCED	EQUITABLE	RPS	AEA
**314**	To perform an observational study to identify which laboratory tests can early diagnose a medication error.	48	46	58	44	46	54	49.5	0.282
**315**	To analyze and identify the root cause of multidrug resistance in the treatment process to create more effective interventions.	58	50	34	53	53	47	49.4	0.353
**316**	To conduct a longitudinal observational study of patient medication non-adherence on health outcomes.	72	50	34	51	43	43	49.0	0.452
**317**	To evaluate the efficacy of generic antibiotics compared to their original patented brand. Do they have the same impact on antibiotic-resistant bacteria in the digestive flora?	64	44	44	46	46	46	48.5	0.282
**318**	To compare the efficacy of generic medication to the original index drug and all other generic forms?	63	46	32	50	46	54	48.5	0.333
**319**	To assess the consequences to the individual's well-being and to their effectiveness when the workplace pursues complete elimination of avoidable harm.	50	50	52	46	48	39	47.6	0.290
**320**	To develop Shared Care Guidelines for selected medicines, to promote safe continuity of care in the community.	61	48	29	48	46	52	47.3	0.329
**321**	To compare generic marking of every individual medication and dosage against existing medication in improving medication safety?	60	48	45	45	40	43	46.9	0.230
**322**	To conduct a study exploring implementation methods of drug classification systems in LMIC.	58	40	39	52	42	46	46.1	0.290
**323**	To conduct a study investigating clinical situations that lie outside the guidelines, is there an increased incidence of unnecessary bridging with heparin or low molecular weight heparin?	60	47	40	55	40	34	45.9	0.353
**324**	To develop clinical guidelines for rarely used drugs and perform audits on use.	59	43	48	44	38	40	45.4	0.317
**325**	Assessing the benefits on patient safety and efficacy of marking expiration month and date on tablets.	62	39	41	53	38	40	45.4	0.349
**326**	To conduct an experimental study investigating the differences in the length of carriage of resistant bacteria, after exposure to a single course of antibiotics.	70	41	40	35	39	37	43.6	0.353
**327**	To develop digital thermometers for use with medicine fridges and freezers.	66	42	23	59	30	40	43.2	0.413
**328**	To identify ways to ensure that the systemic problems (and failings) of medication safety amongst health care professionals will not conflict with the current trend of increasing patient knowledge and awareness.	45	38	47	43	43	40	42.8	0.341
**329**	To investigate the effect on patient safety if medication is infused through central vs peripheral veins.	58	42	26	52	44	27	41.4	0.433
**330**	To research into producing a medicines handbook that classifies medication by disease and patient group, that can be applied to different geographic country contexts.	44	36	43	40	36	45	40.6	0.369
**331**	To conduct an exploratory study on the conditions and regulations needed to adopt the prescription to OTC switch.	52	32	38	43	27	43	39.4	0.282
**332**	To create recommendations to accurately identify a patient which could be applied to different institutional contexts.	50	42	27	48	38	25	38.2	0.329
**333**	To investigate the change in the status of the medication to create reliable processes.	29	31	24	28	28	26	27.4	0.325

RPS – research priority score, AEA – average expert agreement

*Specific scores, ranging from 0-100, are presented for each of the 6 priority-setting criteria: answerability, effectiveness, innovativeness, implementability, potential for burden reduction and equitability. Questions are ranked according to their overall research priority scores (RPS), which also has a maximum theoretical range of 0%-100%. Average expert agreement, which can theoretically range from 25%-100%, is also provided for each question.

The overall three highest-ranking research questions (with RPS 86.7-89.2) focused on:

- Assessing how the incidence of harm due to prescribing errors can be reduced by different interventions in low-resource settings;
- Assessing the prevalence, main factors responsible and effective interventions for preventing severe avoidable medication-related patient harm in low-resource settings through pilot studies;
- Identifying affordable and effective methods of improving medication literacy among patients in resource-limited settings.

**Figure 1:**
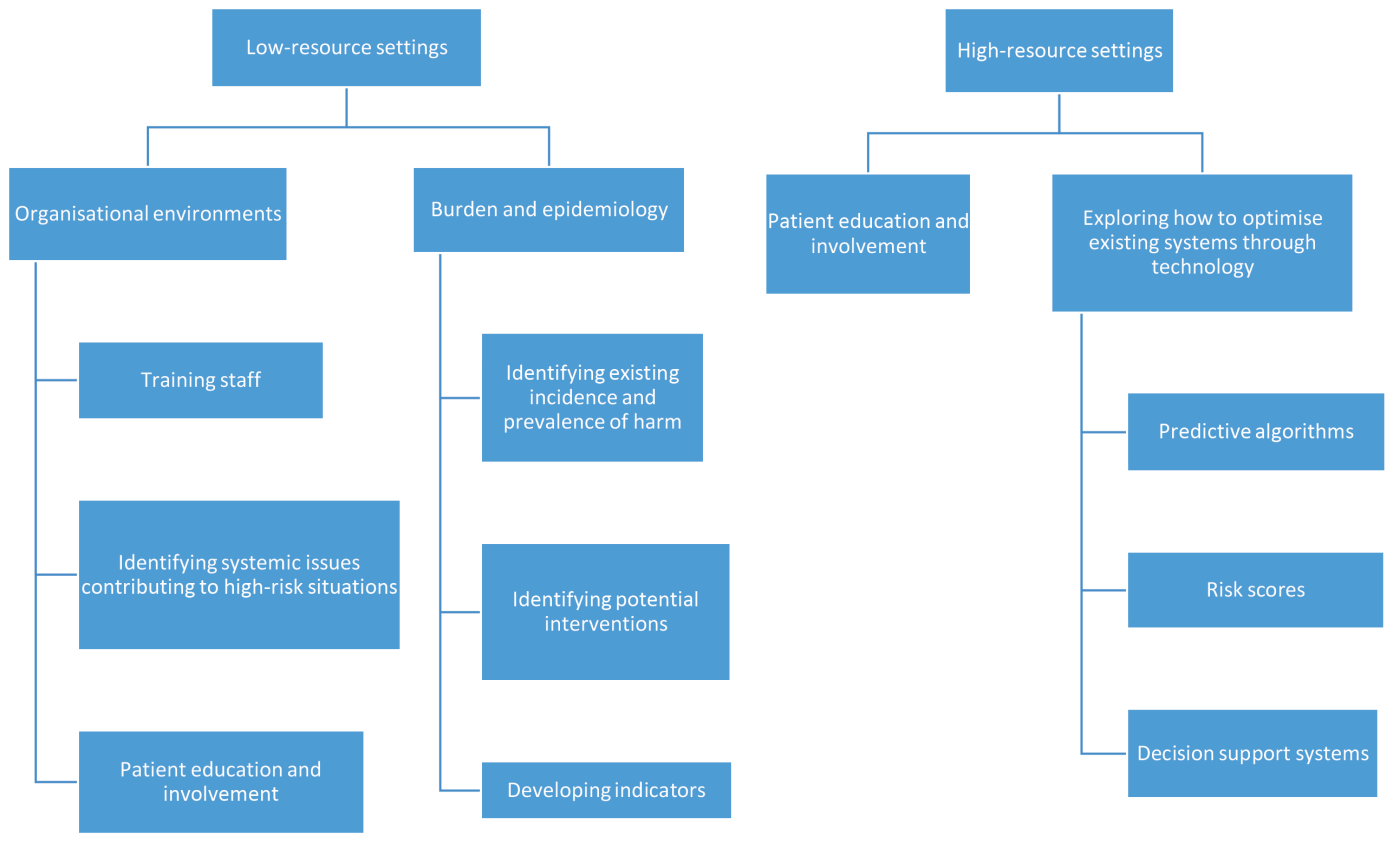
Key priorities for low- and high-resource settings.

**Figure 1** illustrates the key priorities for low- and high-resource settings.

Below, we consider the emerging themes, areas of overlap of priority areas, and discrepancies in more detail.

### Recognised importance of low-resource settings

Our analysis shows that almost half (9 out of 20, 45%) of the top research priorities across experts related to identifying and addressing medication-related harm in low-resource settings (see **Table 1**). The prioritised questions included identifying existing incidence and prevalence of harm, training staff, identifying potential interventions (patient education/involvement, technology, effective medication reconciliation), and developing indicators. Three of the top 10 (30%) priorities identified by experts working in high-resource settings concerned tackling issues in low-resource settings (see **Table 2**).

### Exploring how to optimise existing systems through technology

Investigating how technology can be used to optimise existing efforts aimed at reducing medication-related harm was identified as a priority, particularly in high-resource settings (**Table 2****).** The top 20 overall priorities comprised the development of predictive algorithms and risk scores (see **Table 1**), and included human factors considerations, and information presentation. Of the top 10 priorities identified in high-resource settings (**Table 2**), four (40%) included technology (predictive algorithms and risk scores), technology in low-resource settings, and decision support systems. Pictorial information presentation featured in 20% of identified priorities, but it is unclear if these related to digital systems. Top priorities in low-resource settings did not include technology (**Table 3**).

### Focus on organisational environments, learning and people

Patient education and involvement was mentioned in 25% (5 out of 20) top overall research priorities (**Table 1**). National strategies and policies were also mentioned, but only in 5% of overall research priorities (**Table 1**).

30% of the highest ranking priorities in high-resource settings tackled patient education and involvement, but wider organisational and health system issues did not feature in the top 10 (**Table 2**).

Priorities in low-resource settings focused on wider questions around organisational environments in which issues surrounding medication safety are situated at a facility, system and national level (**Table 3**). Here, the top 10 research questions tackled exploring differences between settings with varying levels of income (30%), identifying systemic issues contributing to high-risk situations (20%), and promoting patient education and involvement (20%).

### Overlap between lowest priority and most divergent perspectives

The research questions with the most divergent scores centred on research topics in specific settings eg, investigating health care-associated infections, antibiotics, generic medicines, over-the-counter (OTC) medicines (**Table 4**). Lowest-ranked research questions focused on single study designs in specific settings and medicines, but also included procedural issues such as identifying high-risk patients (see **Table 5**).

There was substantial overlap between six research priorities that were included in both the 10 research questions (60%) with the most divergent scores and the 20 lowest priority research questions (30%).

## DISCUSSION

Our priority setting exercise has shown that medication safety is a truly global problem. Medicines are inadvertently killing and harming patients on a scale that is unacceptable. When viewed globally, and not from the perspective of some leading health care providers in high-resource countries, there has been relatively little improvement since the subject was first documented. This means that neither research, nor its translation into practice, has played a part in protecting patients. Taking a fundamental look at research priorities in medication safety is a necessary step to create opportunities to transform this situation.

There was clear recognition by the experts who participated in our study of the need for research commissioners and funders to support ideas in scope to serve most of the world's population, but also those that are innovative and with realistic prospects for implementation. At the opposite end of the priority spectrum were ideas either formulated too specifically or too focused on a specific context, or simply not clear enough. The significant overlap between the most controversial and the lowest priority areas suggests that the notion of lowest priority is somewhat contested.

The scorers also were of the view that research must encompass low-resource settings, where the burden of medication-related harm is likely to be highest. Patient education and involvement were judged as priority areas across all care contexts. Priorities in high-resource settings focused on optimising existing systems through technology, whilst in low-resource settings priorities reflected the need for wider organisational changes.

The method we used to prioritise research needs is based on a systematic, well-documented process that is transparent and replicable. It is based on a collective opinion of experts. This eliminates the risk of individual scorers unduly influencing the overall score. We engaged many scorers (42 international experts), so there is a very high degree of confidence (>95%) that the ranking of priorities would not greatly change with a different group of scorers, unless major self-selection bias was present [23]. Differences in the contexts used to score are unlikely to have affected the scoring of most proposed research questions. They could however have had an important effect on proposed ideas that were considered not feasible in low-resource settings. This exercise also efficiently discriminated between a very large number of research questions, with RPS spread over a very wide range (27.4-89.2, see Table S2 in **Online Supplementary Document**).

In comparison to previously conducted CHNRI exercises, this work was characterised by a rather low response rate at all stages of scoring. The initial response rate in a typical CHNRI exercise is around 60%, while the response rate at the second stage tends to be around 50% [16]. The scoring of this study was, compared to other CHNRI exercises, burdensome for scorers, so it is possible that this may have turned away many of the invited scorers. That said, simulations have shown that a sample size of 42 scorers should still result in stable and replicable scores and rankings [22,23]. To support this, research questions that were similar in nature were ranked very closely together on the final list, which is one of the useful indicators of robustness of the CHNRI process.

Another limitation relates to the search strategy and sample characteristics of experts. The search strategy for experts may not be representative as the first-author, with the exception of major trials, is seldom the most experienced author or “expert”. There was also clearly an overrepresentation of participants from high-resource settings (31 out of 42), which is likely to have influenced the results in favour of concerns important to those working in these settings. There was also a potential overrepresentation of pharmacists/pharmacologists (18 out of 42), and medical doctors (11 out of 42).

Our findings provide detailed and actionable recommendations for policymakers and research funders to begin addressing the significant burden associated with medication-related harm internationally and in countries with different levels of resources (see Tables S2-S4 in **Online Supplementary Document**). Furthermore, the CHNRI process offers research funders and policymakers an opportunity to understand potential strengths and weaknesses of many research ideas when evaluated against pre-defined priority-setting criteria. This can lead to a multitude of follow-up actions, depending on individual preferences.

Across countries, research priorities reflected a need to identify incidence/prevalence of harm and associated interventions (particularly in low-resource settings) as a necessary first step to improve medication safety. This is aligned with the current efforts of WHO to identify the prevalence of medication errors in low-resource settings [15]. These settings have received limited attention in the literature [25]. It also reflects issues surrounding a lack of agreement on ways to define and measure medication-related harm [26,27].

Prioritised interventions tackled the alignment of informational requirements of the various stakeholders involved in the medication management process (including the patient). Whilst patient involvement reflects a wider international drive towards the increasingly active role of patients in the management of their own health and illness to improve quality of care [28], patients may also be viewed as part of a wider health information infrastructure surrounding medication-related information which is created and maintained by a wide variety of stakeholders [29].

This infrastructure may include both paper-based and electronic systems, depending on context. For instance, technology-based approaches to optimising existing medication management through secondary uses of data are more immediately relevant to high-resource settings that already have basic technological infrastructures in place [30]. Conversely, identified priorities in low-resource settings show that these countries need to focus on wider organisational changes to allow the emergence of basic informational infrastructures (technology-based or otherwise). This indicates that certain pre-requisites need to be in place before optimisation through technology can be considered [31].

## CONCLUSIONS

Our method has identified and agreed research priorities for improving medication safety globally and by income setting. WHO now plans to work with global, regional and national funding bodies to commission work pursuing these research priorities and through doing so contribute to efforts to reduce the unacceptable burden of medication-related harm.

## Additional material

Online Supplementary Document
