# Supplementary material for: Agreeing on global research priorities for medication safety: an international prioritisation exercise
Source: J Glob Health. 2019 Feb 27;9(1):010422. doi: 10.7189/jogh.09.010422 (PMC6393844; doi:10.7189/jogh.09.010422)
Supplement: Online Supplementary Document [file jogh-09-010422-s001.pdf]

Sheikh at al. J Glob Health 2019; 9:010422

Supplementary Table 1: Characteristics of experts

Number	Gender	HIC/LMIC	Education (if available)
1	Male	HIC (Canada)	MD, MPH
2	Male	HIC (Switzerland)	M.D., FEAPCCT
3	Male	HIC (Saudi Arabia)	
4	Female	HIC (Ireland)	PhD in pharmacy
5	Male	HIC (Israel)	MD
6	Male	HIC/LMIC	MD
7	Female	LMIC (Egypt)	BPharm, Masters in International Health
8	Male	HIC (Spain)	MD, PhD, MS
9	Male	HIC (France)	
10	Male	LMIC (Jordan/Malaysia)	PharmD
11	Male	LMIC (India)	Master's Degree, Hospital & Health Information Management
12	Female	LMIC (Sri Lanka)	MD
13	Male	HIC/LMIC (UK/Kenya)	MPH, MBA,LLB (law)
14	Male	LMIC (Tunisia)	MD
15	Female	LMIC (Iran)	PharmD, MPH
16	Female	LMIC (Ghana)	MPH
17	Male	LMIC (Ghana)	BPharm MSc (epid) PhD
18	Male	LMIC (Malaysia)	PhD in social pharmacy
19	Male	LMIC (Ethiopia)	PhD in pharmacy
20	Male	LMIC (Nigeria)	Pharmacist
21	Female	HIC (Denmark)	PhD in social pharmacy
22	Male	HIC (UK)	LLM (law)
23	Male	HIC/LMIC (Norway/Iran)	Nursing

24	Male	HIC (UK)	PhD in Pharmacology
25	Female	HIC (Finland)	Ph.D in pharmacy
26	Female	HIC (Ireland)	Registered nurse, MSc Healthcare Ethics and Law
27	Female	HIC (Norway)	Ph.D
28	Male	HIC (Spain)	MD
29	Male	HIC (UK)	MRPharmS
30	Male	HIC (Belgium)	Pharmacist
31	Female	HIC (Australia)	PhD, masters in nursing science, MPharm
32	Female	HIC (New Zealand)	MPharm PhD
33	Male	HIC (USA)	PhD
34	Male	HIC (Switzerland)	PhD in public health
35	Female	HIC (Ireland)	Pharmacist
36	Female	HIC (UK)	PhD in pharmacy
37	Female	HIC (UK)	MD
38	Male	HIC (UK)	MD, consultant anaesthetist
39	Female	HIC (US)	MD, Master of Science in Public Health
40	Female	HIC (UK)	Pharmacist
41	Female	HIC (Netherlands)	PhD in pharmacy, MSc epidemiology
42	Female	HIC (USA)	PhD in Pharmacy Administration

Supplementary Table 2: Final ranks for 333 proposed research questions based on the scores from 42 experts in medication safety. Specific scores, ranging from 0-100, are presented for each of the 6 priority-setting criteria: answerability, effectiveness, innovativeness, implementability, potential for burden reduction and equitability. Questions are ranked according to their overall research priority scores (RPS), which also has a maximum theoretical range of 0-100%. Average expert agreement, which can theoretically range from 25-100%, is also provided for each question.

RANK ALL	RESEARCH QUESTION	ANSWERABLE	EFFECTIVE	INNOVATIVE	IMPLEMENTABLE	BURDEN REDUCED	EQUITABLE	RPS	AEA
**1**	To assess how the incidence of harm due to prescribing errors can be reduced by different interventions in low- and middle-income countries.	94	95	74	84	97	91	89.2	0.643
**2**	To assess the prevalence, main factors responsible and the effective interventions for preventing severe avoidable medication related patient harm in resource-limited settings through pilot studies.	90	92	76	85	90	88	86.8	0.575
**3**	To identify affordable and effective methods of improving medication literacy among patients in resource limited settings	91	91	73	89	87	89	86.7	0.615
**4**	To develop a predictive algorithm to identify individuals who are at risk of serious medication-related harm.	88	91	90	79	94	76	86.2	0.742
**5**	To investigate the role of health communication strategies to support patients with limited language proficiency, health literacy and education in taking medications safely.	89	88	73	85	85	95	85.8	0.571
**6**	To assess the impact of increasing the amount of trained human resources to reduce medication errors in low- and middle-income countries	91	87	79	80	90	81	84.6	0.599

**7**	To develop and validate a complexity score (c-score) to identify the patients who are at risk of readmission in 30 days due to medication errors which could be used by pharmacists and physicians.	91	85	79	90	88	72	84.3	0.631
**8**	To improve medication safety for in-patients, through the application of ergonomics and human factors in the organization of the medications flow: order, distribution, stocking, preparation and administration.	92	86	76	86	89	75	83.9	0.575
**9**	To identify the most effective empowerment methods and tools for patients and their caregivers to speak up when they see the potential for medication-related harm, especially applicable to patients in LMICs, as often the most impacted individuals are poorer and less educated.	85	79	84	79	82	94	83.6	0.595
**10**	To develop and validate a complexity score (c-score) for patients in need for de-prescribing which would help the physicians or pharmacists identify the high-risk patients who might develop drug-drug interactions.	91	84	80	86	84	75	83.3	0.563
**11**	To identify and develop globally applicable pictograms for selected high-risk medications which would convey the critically important safety information.	87	82	72	83	88	87	83.1	0.536
**12**	To conduct a study investigating the types of medication-related harm that occur in transitions between hospitals and primary care settings in LMIC.	97	86	63	83	89	80	82.8	0.571

**13**	To create patient knowledge-building tools for medication safety with critical thinking to ensure they are usable for people with low level of literacy, in a reliable format and addressing the role of internet as an information source.	88	83	80	81	78	86	82.6	0.623
**14**	To investigate how technologies could be appropriately implemented and scaled in LMICs to better ensure that drugs are not spoiled, diverted, counterfeited, and that supply chain performance is optimized to avoid stock outs and drug shortages.	83	90	70	83	84	82	82.0	0.452
**15**	To compare the benefits of pictorial information in medication instructions to written instructions alone, in improving medication safety. To what extent, in what contexts and formats is pictorial information most beneficial?	88	78	80	80	76	83	81.0	0.500
**16**	To identify what national strategies and/or policies for medication safety across high-, middle-, and low-income countries exist. What gaps remain in identifying and implementing these prevention strategies/policies?	95	84	63	75	82	85	80.8	0.540
**17**	To evaluate the impact of medication reconciliation in preventing medication errors in low-income countries.	94	85	63	73	85	83	80.6	0.563
**18**	To identify indicators of medication safety that have been utilised in low-resource settings. What is known about their validity, reliability, and feasibility, and what potential indicators should be introduced?	93	80	69	83	78	80	80.6	0.508

**19**	To investigate how to ensure patient safety for patients utilizing oral home-based chemotherapy administration: maximising patient education and monitoring systems.	91	85	76	86	77	69	80.6	0.548
**20**	To identify the reliable easily measured indicators to assess medication safety both at facility level and at national level	88	83	67	86	80	73	79.6	0.540
**21**	To evaluate the effectiveness of the Medication Without Harm Challenge by using Interrupted Time series to assess the implemented policies to prevent medication errors by measuring the situation prior and after (e.g. 6 months before and after) the introduction of the implementation of the policy.	89	84	71	81	79	69	79.0	0.528
**22**	To determine the prevalence and potential clinical outcomes of medication errors in (1) primary care settings, and (2) hospitals in low resource countries.	87	81	70	75	79	80	78.8	0.567
**23**	To identify the most frequent patient-caused medication errors that occur at home and assess what strategies could be implemented.	89	82	77	81	80	64	78.8	0.599
**24**	To identify and create recommendations for the most effective approach to decision support alerts in electronic prescribing systems, the optimum sensitivity and specificity and criteria which should be used to enable prescriber to receive alerts but not receive alert fatigue.	89	83	70	84	83	63	78.7	0.468

**25**	To assess cost-effective methods to keep track of what medicines a patient has taken and is taking, investigate how to record and share patient medication histories across different health infrastructures in order to reduce medication errors leading to medication-related patient harm?	81	85	66	81	85	74	78.7	0.571
**26**	To assess the prevalence of medication errors among patients who self-administer their medication in home setting and assess different methods of patient education as an intervention.	83	80	83	79	79	69	78.6	0.599
**27**	To test the use of a patient decision support system in order to ensure patient safety within self-management of medications in chronic conditions, with the remote support of health professionals and group of peers (patient experts).	85	75	78	80	77	76	78.4	0.480
**28**	To quantify the impact of electronic systems and knowledge-based clinical decision support on reducing medication-related harm in LMICs, by implementing this technology to smart-phones.	87	79	84	78	75	66	78.3	0.508
**29**	To develop and test a core set of medication safety indicators, applicable for different contexts and countries worldwide. The set of core indicators may differ according to income level of country.	86	79	71	76	75	83	78.3	0.544
**30**	To identify pictograms which could be used to teach patients about their medicines	89	72	63	80	82	83	78.2	0.468

**31**	To identify and create new indicators and metrics for medication safety to measure better the impact of medication safety work.	81	82	78	81	77	69	78.2	0.512
**32**	To investigate the effects of different interventions before/during/after the hospital discharge to ensure patients know and understand the information on their medication.	89	82	59	80	82	76	78.0	0.528
**33**	To evaluate the reduction in medication errors at a local level after introducing a program which aims to increase the role of patients in medication safety, engage them in their care and increase patient counselling, in minimizing the incidence of medication-related harm.	84	84	71	80	79	69	77.8	0.563
**34**	To evaluate which tools can be most useful to measure medication safety and determine the impact of measures that are implemented over time	83	83	67	83	81	68	77.6	0.536
**35**	To develop a mobile technology tool for patient and clinician use, aiding appropriate polypharmacy in LMIC.	88	83	79	70	74	71	77.4	0.448
**36**	Can implementing interventions related to barriers to de-prescribing, in patients with multi-morbidity and polypharmacy, minimise morbidity, mortality, reduce financial burden, improve medication adherence leading to enhance patient safety?	79	81	70	80	82	71	77.2	0.560
**37**	To assess how decision support systems can be integrated with patient data, the culture barriers to rational prescribing in resource poor countries and how electronic prescribing systems relate to traditional medicine.	78	84	78	70	79	73	77.1	0.476

**38**	To study the feasibility, impact and scalability of decision support tools to assist clinicians in safe medication prescription practice	92	83	56	77	84	70	76.9	0.528
**39**	To assess the reporting and learning of medication error systems at global and regional level and their impact on system change	90	81	67	74	74	76	76.8	0.591
**40**	To evaluate medication discrepancies, communication, documentation and the role of medication reconciliation across care transitions in preventing medication-related patient harm.	93	89	47	78	88	66	76.8	0.520
**41**	To develop a pilot implementation tool kit to improve medical and health care safety processes, applicable for different contexts and countries worldwide.	77	77	75	75	76	80	76.8	0.496
**42**	To identify certain group of diseases and patient groups that correlate to higher chances of experiencing a medication-related error and would such greatly benefit from conducting routing medication reviews and medication reconciliation.	80	85	61	82	81	72	76.6	0.639
**43**	To assess which aspects of organizational culture and/or context can help to prevent medication-related patient harm.	87	80	66	78	75	73	76.6	0.520
**44**	To examine the impact of the Essential Medicines List in LMIC, to prevent and address inappropriate polypharmacy at transitions of care.	80	73	74	77	73	81	76.4	0.464

**45**	To assess how in resource poor countries/regions, evidence-based medication management systems which cover from prescribing to administering drugs can be implemented, and what organizational and cultural requirements are needed to implement such systems?	77	79	73	68	78	82	76.4	0.492
**46**	To identify strategies to enhance patient communication processes on medication management in hospitals and across transitions of care.	87	77	69	79	78	67	76.3	0.544
**47**	To investigate the impact of addressing high alert medications on morbidity and mortality in two pilot sites, one in LMIC and one HIC.	80	76	65	82	79	74	76.2	0.571
**48**	To research into the utilization of digital health services monitoring large population health records to surveil adverse drug reaction signals.	81	73	83	79	81	60	76.2	0.452
**49**	To examine errors in chemotherapy dosage and/or administration, what organisational changes can be implemented to prevent them from occurring?	90	84	58	84	74	67	76.0	0.536
**50**	What are the most frequent causes of severe, avoidable medication-related harm in high-, middle-, and low-income countries? If this is not known, what steps need to be taken to build and/or strengthen surveillance systems to identify medication-related harm?	82	76	62	77	81	77	76.0	0.532
**51**	To evaluate the economic impact of interventions targeted to reducing medication-related adverse events.	88	82	65	73	75	72	75.8	0.496

**52**	To perform a multi-modal analysis utilising a mixed methods approach assessing human factors, to analyze the most effective medication administration practices to prevent patient harm.	83	80	72	74	78	67	75.7	0.456
**53**	To evaluate the role of human factors, ergonomics and error-proof designing of health care facilities as a medication safety strategy	89	77	73	80	75	59	75.6	0.480
**54**	To conduct a patient-centered study on the effectiveness of medication counseling on decreasing misuse and identifying errors.	84	77	70	78	75	70	75.6	0.520
**55**	To examine the barriers to medication reporting amongst health care professionals in LMIC and what can be done to overcome them.	89	77	69	75	71	72	75.5	0.579
**56**	To investigate identification systems (correct patient, correct route) and functional designs of hospitals to facilitate safe administration of medicines to patients with cognitive impairment.	84	77	57	81	80	74	75.5	0.480
**57**	To conduct a study across institutions identifying system-level barriers to implementation of evidence-based health literacy practices.	88	74	69	70	72	79	75.5	0.460
**58**	To identify globally applicable list of questions patients should know about their medications.	87	78	55	75	74	84	75.3	0.536

**59**	To determine the extent of harm contributed by sub-standard, spurious, falsified and counterfeit medicines (SSFFC) to patient safety. How to reduce the prevalence and incidence of SSFFC medicines in LMIC for better health outcomes?	69	81	60	76	84	82	75.3	0.520
**60**	To investigate the correlations between patient education and engagement with adherence to medication, inappropriate prescriptions and adverse drug events; and to identify which education tools are effective and sustainable.	78	75	74	81	76	68	75.1	0.532
**61**	To evaluate the role of service design and technology in improving safety at transitions of care	80	79	77	76	78	61	75.1	0.512
**62**	To assess how globally data on medication errors and medication related harm is recorded beyond pharmacovigilance and implement a global database which would collect data on medication errors and near misses that especially low- and middle-income countries could report to.	75	73	73	73	72	84	74.9	0.504
**63**	To investigate cost-effective, multidisciplinary practices for optimising patient pharmacotherapy, especially for patients with chronic conditions and multi-morbidity.	83	83	56	79	84	64	74.8	0.563
**64**	To perform a qualitative study on patient's perspective: what is their role in medication management during transitions of care and what do they think it could be? Identify strategies used to engage patients in medication management during transitions of care. What are the barriers and facilitators to these strategies, and to assess the	85	77	69	82	71	65	74.8	0.536

	effectiveness of the identified strategies?								
**65**	To evaluate forms of optimizing drug prescribing in the elderly in collaboration of pharmacists and GPs.	84	84	45	82	82	70	74.8	0.492
**66**	To examine the most frequent cause of hospital admissions in polypharmacy patients. What strategies can be implemented to reduce the risk?	85	83	52	77	87	64	74.7	0.508
**67**	To create a cost-effectiveness study to identify mechanisms that support safe prescribing for elderly, poly-medicated patients.	84	84	60	77	76	68	74.7	0.464
**68**	To identify globally applicable list of minimum questions health care professionals should ask their patients before dispensing or prescribing medications.	89	73	67	80	70	68	74.6	0.520
**69**	To conduct exploratory research into how potential medication safety issues can be predicted in a timely manner, with the use of algorithms based on clinical data.	76	76	91	72	75	57	74.6	0.444
**70**	To perform a mixed-methods study examining the role patients and families play in contributing to medication errors due to non-adherence or over-adherence, and how much of this is due to issues with regards to access or health literacy? What evidence-based interventions exist?	83	78	67	79	70	70	74.5	0.647

**71**	To conduct an observational study examining the handling of high-risk medications across the continuum of care, and to identify strategies to ensure its safe use.	84	80	63	79	78	62	74.3	0.591
**72**	To identify best strategies to avoid inappropriate self-medication with antibiotics.	88	86	48	79	72	73	74.2	0.520
**73**	To design and implement an information campaign aimed at patients to increase patient empowerment in reducing medication-related harm by addressing: transparency and completeness of information to be shared with healthcare providers, storage of medication, medication adherence, medication shifts, possible adverse effects and adverse drug reaction reporting.	80	77	59	80	73	76	74.2	0.552
**74**	To identify effective communication strategies to ensure medication safety in transitions of care (incl. Appropriate medications and education on those medicines)?	91	85	50	77	80	61	73.9	0.484
**75**	To explore novel ways, we can utilise technology (applications and other automation) to reduce adverse medication-related events. What is the role for technology in assisting patient monitoring and correct use of medications?	86	74	68	79	77	59	73.9	0.524
**76**	To implement national level controlling and monitoring of safety of herbal medications.	84	70	57	83	79	71	73.8	0.437

**77**	To identify which remediable human factors are the most important to provide safe prescription and administration of medicines?	88	84	58	80	76	56	73.7	0.484
**78**	To identify factors that influence patient decision-making about polypharmacy, to develop decision aids for patients to help them take part on the discussion of taking multiple medications.	79	71	71	72	71	75	73.5	0.409
**79**	To conduct a cost-effective analysis on interventions to reduce avoidable drug-related hospital admissions.	79	84	54	79	79	65	73.4	0.544
**80**	To investigate what are the most effective and cost-effective interventions carried out at the individual and community-level, to help reduce inappropriate antimicrobial use and reduce antimicrobial resistance.	89	78	50	79	79	66	73.4	0.508
**81**	To increase awareness among regulators and pharmaceutical industry how look-alike containers, poor visibility of key information (e.g. Active pharmaceutical ingredient) can cause medication errors and assess if dispensing only the necessary dose (breaking the fixed package when needed) would increase rational use of medicines.	85	80	54	79	82	60	73.3	0.429
**82**	To examine global legislation for medication safety, what policies are fundamental for assuring and improving medication safety?	88	77	58	70	77	70	73.3	0.500
**83**	To conduct an intervention study on the impact of medication dispensing technology in preventing and/or reducing medication errors.	91	79	53	78	77	63	73.3	0.488

**84**	To conduct a study amongst health practitioners identifying at the practitioner-level barriers to implementation of evidence-based health literacy practices for explaining medications to patients and their families.	79	71	66	73	71	80	73.3	0.472
**85**	To design a patient pathway based monitoring framework and supporting metrics for evaluation of safe prescribing and administration of medicines.	75	75	75	69	76	68	73.0	0.389
**86**	To assess critical points of the medication process for injectable medicines in high-stakes environment, such as ED, anaesthesia, critical care, and develop strategies for improvement.	84	86	53	81	79	54	72.9	0.536
**87**	To develop and evaluate a pilot patient medication recording system based on a cloud platform, accessible to all health care providers in prescribing and reviewing a patient's medication.	83	79	75	67	74	59	72.8	0.520
**88**	To perform a cost-effective analysis on interventions to reduce preventable medication-related hospitalisations, and medication-related falls and injuries in older adults.	84	84	46	74	78	72	72.8	0.599
**89**	To investigate the impact, feasibility and scalability of patient education techniques to promote reconciliation and adherence delivered in a variety of ways including: pharmacy, clinic, telephone, smart phone application.	82	77	72	68	72	65	72.5	0.536
**90**	To create a comparative study, which would assess the effectiveness of standardized, patient carried basic medication list in a developing country compared to more involved and complex tools (e.g. best possible	76	72	68	69	71	78	72.5	0.504

	medication history lists) used in developed countries.								
**91**	To assess and identify the weak links in the medication safety process chain to consolidate the local systems and resolve the occurring difficulties and differences in practice.	77	85	57	74	71	70	72.4	0.468
**92**	What is the impact of current prescribing and medication use patterns during pregnancy on adverse birth outcomes in LMIC?	69	74	52	68	90	80	72.2	0.484
**93**	To conduct epidemiology studies of adverse drug events and medical errors in non-academic tertiary settings such as nursing homes, cancer centres, and community hospitals.	87	69	60	72	74	69	72.0	0.460
**94**	To conduct a multisite study evaluating interventions that have been successful in optimising medication error reporting and learning systems.	86	74	64	74	70	64	71.9	0.504
**95**	To perform a study on nurses administering medicines in long-term care settings, what is the prevalence and root cause of medication administration adverse events?	97	76	44	76	72	67	71.8	0.452
**96**	To evaluate forms of optimizing drug prescribing in the elderly in collaboration of pharmacists and GPs.	82	77	46	79	80	66	71.6	0.540
**97**	To commence a policy research on improving the impact of post-market surveillance systems to optimize patient care, drug effectiveness, and market corrections (drug recalls, etc.), including	83	71	60	77	70	69	71.6	0.429

	an assessment how the post-market surveillance systems differ across all countries and how this affects to medication safety.								
**98**	To examine errors related to look-alike, sound-alike medications that occur at the point of care and identify methods to prevent them.	91	77	48	77	77	58	71.4	0.472
**99**	To build methodological capacity in low- and medium-income countries (LMIC) with the aim of improving pharmaco-epidemiological research practices.	81	69	65	67	71	76	71.4	0.448
**100**	To trial a pilot intervention study of the barcode system for medication in LMIC.	83	75	63	65	72	70	71.3	0.425
**101**	To conduct a pilot study of the implementation of various strategies to reduce the use of potentially dangerous medications amongst elderly patients.	80	81	49	78	75	66	71.3	0.544
**102**	To identify the most effective strategies to enhance patient and caregiver`s engagement in medication safety.	74	74	56	79	78	69	71.3	0.532
**103**	To identify the predictor factors for patient readmission to hospital for medication-related problems in patients with complex conditions.	78	82	61	74	74	59	71.3	0.575
**104**	To identify best strategies to inform the public that taking multiple medications can carry risks.	85	70	56	77	68	72	71.2	0.492

**105**	To identify and evaluate potential strategies into how parents and caregivers can be effectively supported with providing high-risk medications to children.	78	76	63	76	64	69	71.0	0.544
**106**	To investigate methods of strengthening the review system of drug names, to prevent the confusion of look-alike, sound-alike medications.	84	76	46	78	78	62	70.7	0.448
**107**	To design a uniform global standard of labelling for OTC medications and create recommendations for good labelling practice, including use of simple language.	83	74	60	69	72	66	70.6	0.444
**108**	To collect evidence on the impact of patient empowerment, engagement and patient charters on reducing severe, avoidable medication related harm.	73	75	68	77	66	64	70.4	0.508
**109**	To identify a set of best trigger tools to identify a declared or potential adverse effect with high risk medications.	80	76	70	73	70	53	70.4	0.480
**110**	To identify through systematic literature review best medication practices which could be implemented (modified to country context) in other countries?	84	72	52	71	75	69	70.4	0.456
**111**	To implement interventions and WHO recommendations that would ensure the correct dosage and safety of medicines for children.	73	77	44	75	77	76	70.3	0.508
**112**	To investigate best practices to prevent adverse drug reactions for people with previous medication-related allergic reactions and hypersensitivities.	74	78	56	78	79	57	70.3	0.520

**113**	To implement an information campaign which would aim to inform population in in a more conscientious way about traditional medicines and food supplements possible risks and interactions with their usual medication.	85	68	54	76	70	68	70.3	0.425
**114**	To design and evaluate effective interventions designed to improve the uptake of health literacy practices related to medication teaching.	73	68	69	67	69	75	70.2	0.381
**115**	To identify the best practices for outpatient medication reconciliation followed by creating a toolkit out of the best practices (incl. interventions aimed at patients/caregivers, staff, clinics, health systems, and communities).	84	69	58	75	74	62	70.2	0.496
**116**	To identify measures which would enable safer medication-taking practices at home in LMIC.	78	71	60	69	68	74	70.2	0.437
**117**	To investigate what areas of medication safety patients can best empower patients as guardians of safety for their own medication. What strategies can be utilized to promote this role and how can it be implemented?	75	69	66	73	67	71	70.1	0.417
**118**	To conduct a multisite analysis investigating hospital adverse events. What has been the impact of electronic medical record (EMR) systems, the different modes of EMR, and optimal approaches in design and delivery?	85	74	61	65	75	60	69.9	0.393
**119**	To design technology assisted medication administration to reduce medication errors e.g. two-tier level identification.	83	77	55	73	77	55	69.9	0.480

**120**	To assess the effectiveness of different implementation strategies for sustaining and/or scaling up evidence-based interventions to reduce, eliminate or ameliorate medication-related patient harm?	74	74	58	73	73	67	69.8	0.429
**121**	To identify weak points in the design of medication and/or its packaging and develop changes which will help reduce mistakes in medication administration.	83	74	54	73	73	60	69.7	0.397
**122**	To identify high-risk situations applicable globally that should be programmed in all information systems (EHRs, CPOE, e-prescribing).	77	69	66	67	70	68	69.6	0.448
**123**	To conduct a pilot study on elderly patients in a long-term care facility/nursing home, do routine medication reviews reduce the overuse of medications?	90	75	52	78	71	52	69.6	0.421
**124**	To evaluate the use of mobile technology for patients in supporting safer medicines managements, including monitoring of health conditions, education and prompting adherence?	84	70	71	69	69	53	69.5	0.433
**125**	To identify the best and most practical ways to measure preventable medication errors in real time and in patient-centered manner?	68	80	65	69	70	64	69.5	0.476
**126**	To conduct intervention study which utilizes mixed methods approach to transform a unit/hospital with high prevalence of medication errors to a low prevalence.	73	77	60	73	77	57	69.4	0.421

**127**	To identify through literature review most effective interventions for medication safety and evaluate and measure their effectiveness through piloting sites using pre-set indicators.	88	76	47	77	72	58	69.4	0.452
**128**	To investigate how medication-related harm can be measured in feasible way, which requires minimal resources, that can be adapted to existing practices and can be measured ongoing basis.	65	74	72	71	69	64	69.2	0.456
**129**	To conduct a multisite study analyzing the transfer and communication of medication-related information across health care settings, and between patients and providers. What are the weak points, how can current practices be strengthened?	83	66	57	73	73	63	69.2	0.456
**130**	To conduct a longitudinal study on how often systematic medication reviews should be performed in nursing home residents and how medication prescribing should be adjusted as treatment goals shift form preventive/curative to palliative/comforting care.	91	71	61	70	66	56	69.1	0.389
**131**	To assess cost-effective ways to ensure medication compliance and investigate the role of mobile technology/assistive technology, to increase compliance in resource poor settings.	78	63	68	77	64	64	69.1	0.540
**132**	To investigate the reliability of medication safety indicators and tools in integrated care settings.	81	73	65	75	66	55	69.0	0.429

**133**	To create effective techniques in medication error disclosure between health care professionals and to the patient, including legal statutes that can promote transparency.	77	70	64	70	71	62	68.9	0.389
**134**	How to adapt patient safety indicators obtained from routinely collected data to different contexts and countries worldwide?	65	68	68	69	71	72	68.8	0.444
**135**	To investigate the role of pharmacist's intervention in reducing medication errors through prospective cohort study. In which ways can the role of pharmacists be expanded to be actively involved in the medication prescribing process.	84	77	38	76	79	58	68.7	0.460
**136**	To examine each step of the medication process, what is the impact of interruptions in different stages and what implements can be made for improvement?	75	73	56	77	73	56	68.5	0.425
**137**	To conduct a pilot intervention to develop a sharing platform among hospitals for describing errors and solutions.	82	71	47	73	69	68	68.5	0.484
**138**	To investigate the response to opioids in middle-income countries: is the rise of opioid misuse, abuse, dependence associated with the increased availability of extended-release opioids.	76	60	66	70	70	69	68.5	0.393
**139**	To perform a study on the role of pharmacists in elderly patients transitions of care. What is their role, how can it be improved and what is the impact?	86	75	47	72	73	58	68.4	0.468

**140**	To identify and evaluate the primary national systems and/or strategies in place for identifying the burden and scope of medication-related harm in high-, middle-, and low-income countries?	84	64	50	67	73	73	68.4	0.464
**141**	To identify methods to reduce inappropriate prescribing of antibiotics, and to develop strategies to minimize use.	79	75	40	82	76	59	68.3	0.528
**142**	To perform a scoping review on devices/aids available to patients for reducing medication errors.	81	69	64	71	67	57	68.3	0.456
**143**	To perform a cross-sectional study across primary care facilities assessing medication safety and exploring strategies in which it can be improved.	80	70	45	81	72	61	68.2	0.476
**144**	To investigate medication review process, which components are vital during medication review to ensure appropriate prescribing for patients with multiple morbidities.	72	73	59	71	74	60	68.2	0.528
**145**	To conduct and evaluate the impact of medication reconciliation practices during transitions of care (e.g. post-hospital discharge).	86	72	39	71	82	59	68.1	0.548
**146**	To apply modelling techniques on the intervention of specific medication problems in specific regions/countries, to ensure an effective and efficient allocation of resources.	66	66	69	61	66	79	68.0	0.377

**147**	To assess how to design the best environment for learning from medication-related incidents	70	69	72	67	69	61	67.9	0.464
**148**	To investigate the problem of fake medicines and their international trade from surveillance, prevention, and interdiction standpoint to get a better and more resolute idea of the true scope and prevalence of substandard and falsified medicines.	61	73	59	67	76	72	67.9	0.460
**149**	To identify and build a platform which would allow patients to access their up-to-date medication list in all times regardless of the physical location.	76	76	56	67	76	56	67.8	0.512
**150**	To assess the health outcomes in patients with chronic conditions who receive deliberate sensitization about possible side effects, adverse effects, interactions during their visits to the clinic against another group which receives the normal (no intervention) counseling.	81	66	63	68	70	60	67.8	0.409
**151**	To assess the role of regulators, manufacturers and the pharmaceutical industry in reducing medication related harm.	73	70	57	67	70	69	67.5	0.429
**152**	To perform an observational multisite study (across tertiary, long-term care, and primary care facilities) to assess how harm from drug interactions (including traditional and complementary medicines) are averted.	88	67	57	66	67	60	67.4	0.413
**153**	To assess the medication safety practices in long-term care facilities	93	71	34	72	73	60	67.3	0.429

**154**	To test through pilot sites which are most fruitful and powerful combinations of practices, processes, and tools to prevent avoidable medication-related harm.	71	71	56	71	73	61	67.3	0.452
**155**	To create an evaluation framework that measures improvement initiatives so that those can be compared (by how effective, efficient, timely, patient-centred, equitable and safe each intervention is).	76	68	63	70	64	62	67.2	0.405
**156**	To assess the effectiveness of implementing a tool in detecting medication errors and harm in primary care?	70	75	45	73	70	70	67.2	0.468
**157**	To assess the human factors which affect the decision-making process during prescribing, to teach best practices to doctors.	79	69	66	67	64	57	67.1	0.476
**158**	To identify which human factors are either involved in or contribute to medication-related patient harm	79	70	51	73	71	59	67.1	0.480
**159**	To design and develop sustainable, childproof and tamperproof containers which would ensure there is no risk to take dose higher than intended (e.g. Impossible to dispense more than 10 ml before realigning it).	76	69	60	68	67	62	67.1	0.413
**160**	To investigate incidents of medication-related patient harm, what role and to what extent does technology and technical complexity contribute?	78	66	68	68	70	52	67.0	0.421

**161**	To investigate the prevalence of adverse drug events and medication errors contributed by extemporaneous compounding medications for children, due to limited access to safe and effective medicines.	66	70	63	64	68	70	66.8	0.520
**162**	To identify cost-effective, sustainable methods for community-based promotion of medication safety interventions.	72	75	65	67	69	53	66.8	0.393
**163**	To identify the best and most cost-effective ways to transfer medication records in transitions of care	78	76	55	67	73	52	66.8	0.440
**164**	To develop standard protocols for the preparation, administration, monitoring of insulin in acute care settings, for application internationally.	77	70	44	71	71	67	66.7	0.492
**165**	To investigate how having an understanding about health literacy can contribute towards ways that practitioners give information to patients about their medications.	72	68	52	65	67	76	66.6	0.405
**166**	To assess how innovative models of pharmaceutical care and diffusion of technology can be used to reduce medication related harm.	75	68	55	69	67	65	66.6	0.401
**167**	To assess the benefits technology can play in reducing medication errors caused by verbal orders, during transition of care and in engaging and educating patients on their medication.	80	69	65	63	76	47	66.5	0.413
**168**	To conduct a study into patient-user strategies, such as e-health and patient information, as facilitators for safe medication use and medication adherence.	78	64	68	73	62	54	66.4	0.496

**169**	To evaluate the prevalence of unnecessary medications and food supplements, drug-drug interactions and drug-disease interactions among patients who take multiple medications.	77	76	45	66	76	58	66.4	0.425
**170**	To analyze the organizational and functional health care workforce: what can be better configured, developed, and managed to promote medication safety?	73	70	56	71	69	59	66.3	0.440
**171**	To assess how different interventions (including monitoring systems) reduce incidence of harm due to drug-drug interaction in poly-medicated elderly patients.	78	73	53	71	69	53	66.3	0.397
**172**	To examine the role of Artificial Intelligence in reducing medication-related patient harm from the perspective of multiple stakeholders including clinicians, patients, consumers and administrators.	75	64	78	62	67	52	66.2	0.341
**173**	To assess the global prevalence, burden and geographical variation of serious medication related harm through a systematic literature review.	88	65	51	67	61	64	66.1	0.484
**174**	To critically evaluate procedures in the transfer of medication process to ensure safe inter-unit transfer.	77	73	45	66	73	63	66.1	0.448

**175**	To develop safe self-administration models for patients or care takers with chronic diseases to self-administer their medication in hospital and home settings.	73	73	61	62	65	61	65.9	0.444
**176**	To conduct a multisite workplace analysis to determine required staffing levels to achieve minimal medication errors.	79	71	66	64	69	47	65.8	0.409
**177**	To apply global standards on packaging and labelling to have uniformed place and way how and where the best before date and strength of the medicine is placed in the product.	80	69	55	66	66	59	65.8	0.357
**178**	To identify which factors contribute to administration errors where medication is not given in nursing homes and care homes	80	67	46	73	69	59	65.6	0.365
**179**	To conduct an observational study in hospitals that systematically conducts medication reconciliation at discharge. How many adverse medication-related events, re-hospitalizations and deaths are avoided?	85	74	43	64	74	54	65.6	0.520
**180**	To examine how the role of health care professionals can be strengthened to ensure appropriate polypharmacy in the elderly.	67	64	53	71	71	67	65.5	0.452
**181**	To reduce harm related to interactions by supporting creation of an app which would help prescribers in reducing the risk of interactions, supporting in decision making, offering possible alternative options, such as deprescribing, while considering patient's pathologies.	78	70	61	68	67	48	65.4	0.437

**182**	To assess how health care professionals can prevent drug interactions (drug-drug, drug-disease) in a simple and applicable way during daily working	78	72	37	75	69	61	65.4	0.476
**183**	To identify the impact of the availability of the Children`s Medicines List on the unmet needs of Children`s Medicines in Sub-Saharan Africa.	73	63	60	62	60	73	65.3	0.325
**184**	To investigate the change in medication safety practice after presenting medication error root cause analysis to healthcare professionals.	80	69	55	68	64	56	65.2	0.452
**185**	To explore ways to increase multi-professional team work and build respectful environment where teams recognize and respect the skills of each member in ensuring medication safety.	72	64	71	67	67	50	65.1	0.433
**186**	To identify and assess the best ways and tools for health care professionals to provide effective information about safe use of medications to patients and caregivers.	72	74	45	71	67	60	65.1	0.464
**187**	To assess the influence of social determinants of health on adherence to medicines among patients with chronic diseases.	75	63	43	71	56	82	65.0	0.516
**188**	To identify through pilot studies best practices on how to make health care facilities and health care providers accept the existing essential drug lists; and accept the use of Standard Treatment Guidelines in countries where they exist and create them where they do not exist to ensure safe, rational use of medicines.	70	68	46	70	63	72	65.0	0.409

**189**	To establish the scale and burden of inappropriate non-evidence-based use of antipsychotic and antidepressants.	80	70	56	63	66	55	65.0	0.393
**190**	To investigate what are the specific barriers to address appropriate polypharmacy through interprofessional working in low-, middle- and high-income countries.	73	67	54	61	67	68	64.9	0.393
**191**	To identify gaps in deprescribing potentially inappropriate medicines in older people.	72	67	54	71	71	55	64.9	0.373
**192**	To review current prescribing guidelines and develop strategies to reduce overprescribing, particularly for opioids.	80	74	39	71	71	54	64.9	0.488
**193**	To identify the special considerations of medication safety in emergencies and create key policy recommendations on reduction of medication-related harm within the overall cycle of crises management.	73	66	61	64	67	58	64.8	0.393
**194**	To investigate how to increase public reporting of health care associated infections and increase rewarding/giving incentives to healthcare facilities which meet quality and safety standards.	83	61	52	64	65	63	64.7	0.345
**195**	To undertake exploratory research on the feasible implementation of a feedback system for physician prescribing practices.	70	68	66	70	66	49	64.7	0.437

**196**	To identify methods of monitoring patient medication adherence and develop evidence-based practices to improve adherence, reduce patient harm and improve outcomes.	82	78	38	72	68	50	64.6	0.540
**197**	To evaluate which strategies work best to ensure the accuracy of a patients` medication management across the continuum of care.	69	72	46	68	73	61	64.6	0.456
**198**	To conduct exploratory research on emerging technologies to prevent medication events from occurring, available for use in the patient's home and/or long-term care settings.	68	66	68	63	68	55	64.5	0.397
**199**	To assess the prevalence of drug-drug interactions where one of the interacting medicines is prescription medicine and the other is non-prescription medicine.	81	66	54	69	57	60	64.5	0.377
**200**	To form and divulge basic notions about antibiotics, their uses, operations and sequential effects for patients to understand in what moments it is correct to prescribe them and the importance of following the guidelines recommended by their physician.	81	68	33	70	70	66	64.5	0.468
**201**	To create instructions for countries and institutions to help them to set up a good interdisciplinary concertation/coordination to avoid drug related errors/events.	73	67	53	64	65	65	64.4	0.421
**202**	To design a patient pathway based monitoring framework and supporting metrics for evaluation of safe prescribing and administration of medicines.	64	67	60	61	68	65	64.2	0.373

**203**	To design supporting tools for health care professionals which help to prescribe and dispense appropriate medications for old people	62	66	45	72	74	65	63.9	0.429
**204**	To identify the challenges involving the patient and their caregivers in the campaign to improve medication safety, and what resources and support is required?	72	65	58	63	65	61	63.8	0.389
**205**	To investigate what percentage of medication errors are due to similarities in medication appearance.	76	68	48	70	67	53	63.8	0.468
**206**	To research into the development of novel electronic health record data entry methods, to facilitate accuracy and reduce errors.	69	62	61	69	68	53	63.7	0.413
**207**	To evaluate whether expert patient engagement in health systems can reduce severe, avoidable medication-related patient harm.	71	63	65	69	57	56	63.6	0.365
**208**	To perform a study evaluating the strategies to improve patient monitoring of adverse effects of medication.	78	68	44	66	68	57	63.5	0.425
**209**	To create guidelines for safer use of paracetamol (acetaminophen) in children to address safe prescribing, safe and clear dosing schedule and safe dispensing and administration.	84	68	37	72	57	61	63.2	0.556
**210**	To conduct a study investigating the impact of procurement based on clinical efficacy and safety, with the use of longitudinal data analytics thereby optimising benefits and minimising harm.	66	69	64	62	65	54	63.2	0.294

**211**	To assess the lack of communication in transitions of care (i.e. at hospital discharge) by conducting an observational and interventional study?	85	65	38	70	68	53	63.1	0.464
**212**	To evaluate the current electronic prescribing process and pilot a redesign on indication and decision supporting drug of choice.	69	65	62	69	65	48	63.0	0.385
**213**	To identify the challenges and opportunities for health care professionals and the health care system to improve medication safety in transitions of care.	72	62	45	68	64	66	63.0	0.421
**214**	To perform a cost-effective analysis on interventions to improve medication adherence to evidence-based cardiovascular pharmacotherapy and other chronic conditions.	76	72	43	74	56	56	62.9	0.476
**215**	To perform a study investigating the underlying causes, extent, and the consequences of a lack of communication between different healthcare professionals and between healthcare professionals and their patients.	76	67	53	61	62	58	62.9	0.401
**216**	To evaluate if there is a difference in factors which contribute to medication errors depending on the level of health care (primary, secondary, tertiary) and health care system.	80	59	58	61	59	60	62.9	0.413
**217**	To assess the nature and frequency of off-label medication use contributing to medication-related harm.	80	70	57	58	57	55	62.7	0.393

**218**	To identify collaborative team practices utilised by health care professionals to ensure appropriate drug monitoring and patient involvement?	73	66	55	63	68	52	62.7	0.381
**219**	To examine ambulatory practice prescribing, which types of medications, or combinations of medications, have a high risk of safety problems in patients.	70	68	47	70	67	54	62.7	0.488
**220**	To investigate strategies to enhance patient engagement and better use patient information, to be applicable and achievable for patients across all income-level countries.	59	61	57	63	65	71	62.6	0.381
**221**	To evaluate the implementation of QR coding for all medicines in their respective market and assess the public awareness of the need to verify the authenticity of medicines in countries which have implemented the QR coding.	75	61	65	61	62	52	62.6	0.345
**222**	To conduct an assessment on compliance and the reduced risk of adverse drug events in patients taking novel oral anticoagulants in lieu of warfarin.	77	66	42	77	63	50	62.4	0.456
**223**	To conduct a study investigating patient stock-piling of medications and conditions of their storage, their beliefs related to use of medicines and sources of medication information.	82	55	61	62	59	56	62.4	0.425
**224**	To conduct an observational study to identify critical laboratory tests, which if incorrect, may lead to a higher risk of medication errors occurring.	73	67	60	58	58	58	62.2	0.361

**225**	To conduct qualitative research on administrators and other managerial decision-makers addressing accountability on the decisions they make that create medication-related harm.	74	60	67	59	57	55	62.1	0.381
**226**	To conduct an intervention study enhancing patient involvement with their prescriber, does this empower patients to raise medication concerns promptly?	73	64	58	67	59	52	62.1	0.397
**227**	To perform an intervention study evaluating whether the provision on information about patient therapy to community pharmacists leads to a decrease in medication errors.	77	65	50	58	63	58	61.9	0.385
**228**	To assess the magnitude of severe drug interactions due to poly-pharmacy in elderly.	73	68	44	65	65	57	61.9	0.425
**229**	To examine incidents of product contamination and drug mislabeling. What techniques can be utilised to improve identification?	73	66	47	65	62	57	61.7	0.389
**230**	To conduct a study within long-term care/nursing home facilities, examining the surveillance and reporting systems of adverse drug events and patient harm due to atypical antipsychotics.	82	60	46	64	63	55	61.7	0.357
**231**	To study the feasibility, impact and scalability of awareness among patients and clinicians, that low kidney function as a major risk factor for medication-related patient safety events, delivered in a variety of ways - pharmacy, clinic, telephone, smart phone application?	69	59	59	65	62	52	61.1	0.393

**232**	To perform a qualitative study investigating what are the perceived causes of unsafe medication practices.	85	62	50	64	58	49	61.0	0.429
**233**	To address the key issues in reconciling prescriptions and administration across settings through a pilot study.	85	62	38	67	63	52	61.0	0.468
**234**	To measure which is the most sensitive moment, weekday and time of the day for occurrence of medication errors: ordering, prescribing, preparing, dispensing, administering or monitoring?	76	63	49	61	63	53	60.9	0.429
**235**	To perform a qualitative cross-professional study that addresses how to optimise the role of the pharmacist in medical teams, medication administration and delivery.	77	62	48	61	63	53	60.9	0.393
**236**	To identify strategies to increase adverse drug event reporting/post marketing surveillance amongst physicians.	75	72	38	68	61	51	60.8	0.516
**237**	To identify medications that requires invasive laboratory monitoring (e.g. blood tests, ECG) and validating a standard recommendation.	79	59	47	69	68	42	60.7	0.425
**238**	To determine the incidence of antibiotics obtained without prescription or from earlier consultation by a physician, and how information technologies can reduce risky behaviours in LMIC.	71	60	53	58	66	55	60.6	0.361

**239**	To assess the clinical significance of drug interactions based on recent studies and creating evidence-based strategies in deprescribing.	74	59	45	67	61	58	60.6	0.421
**240**	To investigate the relationship between the increased availability of prescription opioids and increased illicit drug use.	79	58	55	53	60	58	60.6	0.341
**241**	To conduct exploratory research expanding the role of nurses to take on leadership roles in preventing medication-related patient harm.	72	62	50	67	61	52	60.6	0.353
**242**	To develop and validate models focused on aspects of hospital layout and healthcare worker/patient flow to reduce HAIs.	73	54	55	66	64	52	60.6	0.282
**243**	To identify what strategies practitioners can apply individually to reduce medication errors.	76	66	48	66	56	52	60.5	0.401
**244**	To ascertain and develop the role of clinical informaticians to bridge the gap between health IT design implementation and healthcare professionals.	70	56	65	62	58	52	60.4	0.389
**245**	To assess the occurrence rate, patterns and trends of medication errors in communities across countries.	76	61	41	58	58	69	60.4	0.425
**246**	To conduct a study on developing valid and reliable measures of patient engagement.	69	65	53	65	53	57	60.2	0.440

**247**	To examine the impact of using external, non-biased safety testing companies, in evaluating the risks associated with potential brand names.	74	60	60	63	58	46	60.1	0.341
**248**	To raise awareness amongst Ministries of Health and healthcare leaders in developing countries to highlight the importance of medication safety in primary care.	70	58	45	61	60	65	59.9	0.401
**249**	To conduct a qualitative study for patients, what are their priorities for reducing the risk of medication-related harm?	76	55	58	68	49	54	59.7	0.405
**250**	To assess the effectiveness of annual meetings with community pharmacist, to discuss which medicines (incl. OTC medications) the patient is taking and how and when they are taking them, can reduce medication-related harm among patients who take multiple medications.	79	56	55	58	58	52	59.6	0.373
**251**	To assess in institutional level the obstacles and facilitators of adverse drug event reporting to increase the reporting	88	58	49	65	50	47	59.5	0.421
**252**	To assess practices which can increase or decrease errors in anticoagulant therapy of patients who have transitions of care between secondary and primary care.	70	69	41	59	64	54	59.4	0.369
**253**	To identify strategies to improve compliance of medication in patients, caregivers and health professionals. What are effective newly developed tools for medication safety?	69	54	51	61	67	54	59.4	0.440

**254**	To investigate which quality improvement factors also reduce medication-related harm.	60	63	52	61	61	59	59.4	0.321
**255**	To conduct a qualitative study seeking to examine how to enhance patient engagement amongst patients and their family, to improve medication safety.	78	56	45	63	53	61	59.4	0.401
**256**	To create a study assessing long-term adherence and compliance of patients who have complex multi-medication management, by looking from both the perspective of the patient and health care professional.	77	63	40	66	56	54	59.2	0.512
**257**	To identify the most frequent diagnostic errors and implement practices to prevent them.	68	70	41	59	63	55	59.2	0.401
**258**	What strategies can be implemented to reduce the overuse of medications with poor effectiveness in terminally ill cancer patients?	68	60	52	69	53	53	59.1	0.385
**259**	To identify and examine different types of surveillance programmes utilised to monitor medications with the potential for abuse.	77	54	59	64	57	45	59.1	0.341
**260**	To assess improvements in medication safety after introducing restrictions on drug dispensing (requirement for a valid prescription).	72	64	48	57	60	53	59.0	0.349
**261**	To assess patient attitudes and behaviours towards use and storage of medications in relation to health outcomes.	83	50	50	67	53	50	58.9	0.361

**262**	To develop and evaluate a system that monitors for the incompatibility of chemotherapy drugs with medications prescribed for other comorbidities, to reduce unwanted synergies.	75	66	36	64	66	47	58.8	0.369
**263**	To investigate the prescribing indication for opioids in middle- or third-world countries, has liberal prescribing for end-of-life care transitioned into use for non-cancer pain management.	75	54	54	52	58	61	58.7	0.298
**264**	To create a study comparing effectiveness of two systems, one where medication errors and adverse drug reactions are reported to different entity/portal, and second where these two are combined and the approach is integrated.	81	55	65	55	48	47	58.6	0.365
**265**	To conduct an analysis on what hinders the voluntary reporting of medication errors even when guidelines exist.	79	59	47	63	56	47	58.4	0.429
**266**	To assess through a pilot study if each time patient interacts with health care system the health care professionals would be obligated to look at least the past three visits back would reduce medication-related harm and help health care professionals to recognize medication-related harm.	70	57	67	52	57	47	58.2	0.389
**267**	To evaluate effectiveness of treatments used to manage extravasation of intravenous medications to minimize medication related harm.	68	70	41	61	63	45	58.1	0.401
**268**	To investigate the impact of changing the package on the uptake of safe medication practices.	72	54	56	57	57	54	58.1	0.302

**269**	To conduct a study examining the role of individual health professionals and inter-professional collaboration, who is accountable for ensuring medication safety?	80	60	52	55	48	54	58.1	0.345
**270**	To conduct an intervention study to determine whether handling of intravenous fluids alike medications (with protected access and patient-specific details) will reduce medication-related harm.	72	61	44	62	58	52	58.0	0.365
**271**	To evaluate incidents of inappropriate polypharmacy and develop strategies to reduce harm and improve patient health outcomes.	67	64	38	59	66	53	57.9	0.393
**272**	To investigate the global burden of opioid over-doses and related deaths.	87	59	37	47	57	62	57.9	0.381
**273**	To conduct exploratory research on how pharmaceutical professionals can adopt medication safety as their principal core societal role.	67	55	57	55	55	54	57.3	0.349
**274**	To identify methods to ensure that each patient is taking the correct medication.	61	63	35	60	65	60	57.2	0.389
**275**	To conduct a multi-professional study implementing different interventions to investigate the effect on reducing medication errors.	63	68	45	57	61	50	57.1	0.333
**276**	To implement data analytics to reduce medication related patient harm.	61	63	53	67	58	40	57.1	0.321

**277**	To conduct a study assessing prescribing of non-approved medication and off-label use; and to investigate methods to reduce.	66	65	52	51	55	55	57.1	0.369
**278**	To examine whether the involvement of Health IT is functioning as expected to improve medication safety.	73	63	50	55	55	43	56.5	0.337
**279**	To conduct a study on enhancing the role of pharmacists in multidisciplinary collaboration with prescribers. What are the attitudes of pharmacists and prescribers, how will collaboration best be utilised?	77	60	38	56	58	50	56.5	0.377
**280**	To perform a study identifying medications (and/or combination of medications), that can potentially lead to acute kidney injury in high-risk critically ill patients.	74	59	31	61	61	53	56.4	0.444
**281**	To identify the role of different actors in health care in reducing global incidence of medication related adverse events.	70	59	44	55	55	55	56.2	0.389
**282**	To investigate patient access and use of online medication record systems (such as OpenNotes) and medication schedules.	75	56	63	52	53	37	56.1	0.361
**283**	To perform a study targeting health care prescribers, addressing how to promote effective communication and collaboration amongst other prescribing providers.	66	57	43	59	59	50	55.6	0.313
**284**	To conduct a study identifying what measures are required to develop the health care team`s understanding towards the Standard Protocol of Medicine Reconciliation.	67	57	46	57	55	52	55.6	0.313

**285**	To critically evaluate implementation of a standardized opioid tolerance assessment and documentation prior to administration.	78	52	50	59	54	41	55.4	0.325
**286**	To perform a qualitative study within the health care workforce focusing on what style of leader health care professionals desire to prevent avoidable medication-related harm?	71	53	61	48	52	47	55.4	0.393
**287**	To conduct research into the development of expert systems encompassing a wide scope of patient information (including age, gender, genetic makeup, laboratory tests), to aid as a clinical decision support.	54	56	54	54	61	54	55.2	0.278
**288**	To identify the enablers and barriers to the routine clinical recognition, diagnosis and recording of serious medication-related harm.	73	55	47	57	55	45	55.1	0.345
**289**	To systematically evaluate the most appropriate devices to deliver intravenous medications assessing the risk of complications, and to additionally investigate.	66	58	48	61	57	40	55.1	0.385
**290**	To gather evidence about the clinically important role of pharmacists in medication safety.	70	58	30	56	62	53	54.7	0.413

**291**	To assess various human factors, such as level of informance on medications, as variates to measure which human factors in patients and health care professionals effect prevalence of medication errors.	56	52	46	57	61	54	54.4	0.298
**292**	How does operationalization of 'resilience' in the health care system affect medication safety?	66	50	72	48	52	38	54.3	0.310
**293**	To investigate and develop model success stories utilising health IT.	69	52	52	55	50	48	54.2	0.345
**294**	To perform qualitative ethnographic research exploring health care staff perspectives on safety and the role they play.	72	50	57	55	43	47	53.9	0.345
**295**	To assess the risk of polypharmacy for individual patients and develop cost-effective solutions.	60	63	41	48	63	47	53.7	0.353
**296**	To perform a qualitative study on perceptions of community pharmacists towards the evaluation of knowledge and skills related to safe medication practice.	84	48	38	52	52	48	53.6	0.361
**297**	To assess the efficacy of antibiotic delivery by the per lingual route, what is the impact on the gut flora and contribution to antimicrobial resistance?	70	54	54	46	52	44	53.3	0.321
**298**	To assess through an observational study how adverse drug events happen and implement interventions to prevent them from occurring.	59	61	38	59	61	42	53.2	0.460

**299**	To assess nurses' skills to do dose calculations after absence from work and compare the drug calculation skills of recent nursing students to experienced nurses who have just came back to work from leave which length has been predetermined to be significant enough (e.g. in maternity leave).	80	45	52	55	50	36	52.9	0.337
**300**	To determine factors that drive spread of HAIs and investigate new approaches that minimize the role of the healthcare environment in the spread of germs.	68	58	39	56	50	44	52.5	0.282
**301**	To conduct a multisite/multimodal study evaluating the role and impact of utilising digital methods in the health care process to reduce medication errors.	61	53	47	58	52	44	52.5	0.317
**302**	To conduct exploratory research on new methods to improve user-centred design and technology.	61	46	57	56	52	42	52.4	0.302
**303**	To conduct a study designed to investigate the intentional non-adherence of medication.	64	49	49	49	53	50	52.1	0.373
**304**	To assess the role of nurses in preventing drug-drug interactions.	72	49	42	53	50	44	51.6	0.381
**305**	To conduct an intervention study in hospital emergency departments evaluating the effectiveness of strategies (organizational/functional) to reduce delay and overcrowding.	71	50	32	54	43	59	51.5	0.317

**306**	To create up-to-date recommendations for safe and good prescribing practices.	59	60	33	54	54	47	51.1	0.405
**307**	To identify which elements globally in robust health systems, help to avert medication related harm	61	53	39	50	52	48	50.7	0.321
**308**	To undertake an examination of health policy analyses in LMIC, are the measures sufficient?	63	48	38	52	50	52	50.6	0.294
**309**	To assess the benefits and risks of medications affecting the central nervous system, such as antipsychotics, antidepressants, anticonvulsants, benzodiazepines, in patients with complex conditions such as ADHD, substance abuse/misuse, and chronic pain.	60	59	27	47	59	50	50.2	0.397
**310**	To assess the required global standards required to be in place before introducing a new digital system to any market.	63	46	50	54	44	43	50.0	0.302
**311**	To identify through pilot studies ways to prevent inappropriate use and over-prescribing due to financial incentives to physicians.	55	50	56	45	44	48	49.7	0.329
**312**	To perform a mixed method study examining the role of pharmacists and non-physicians in enhancing medication safety.	67	53	33	53	47	45	49.7	0.349

**313**	To investigate the impact of personalised medicines.	52	48	54	46	46	52	49.7	0.298
**314**	To perform an observational study to identify which laboratory tests can early diagnose a medication error.	48	46	58	44	46	54	49.5	0.282
**315**	To analyze and identify the root cause of multidrug resistance in the treatment process to create more effective interventions.	58	50	34	53	53	47	49.4	0.353
**316**	To conduct a longitudinal observational study of patient medication non-adherence on health outcomes.	72	50	34	51	43	43	49.0	0.452
**317**	To evaluate the efficacy of generic antibiotics compared to their original patented brand. Do they have the same impact on antibiotic-resistant bacteria in the digestive flora?	64	44	44	46	46	46	48.5	0.282
**318**	To compare the efficacy of generic medication to the original index drug and all other generic forms?	63	46	32	50	46	54	48.5	0.333
**319**	To assess the consequences to the individual's well-being and to their effectiveness when the workplace pursues complete elimination of avoidable harm.	50	50	52	46	48	39	47.6	0.290
**320**	To develop Shared Care Guidelines for selected medicines, to promote safe continuity of care in the community.	61	48	29	48	46	52	47.3	0.329

**321**	To compare generic marking of every individual medication and dosage against existing medication in improving medication safety?	60	48	45	45	40	43	46.9	0.230
**322**	To conduct a study exploring implementation methods of drug classification systems in LMIC.	58	40	39	52	42	46	46.1	0.290
**323**	To conduct a study investigating clinical situations that lie outside the guidelines, is there an increased incidence of unnecessary bridging with heparin or low molecular weight heparin?	60	47	40	55	40	34	45.9	0.353
**324**	To develop clinical guidelines for rarely used drugs and perform audits on use.	59	43	48	44	38	40	45.4	0.317
**325**	Assessing the benefits on patient safety and efficacy of marking expiration month and date on tablets.	62	39	41	53	38	40	45.4	0.349
**326**	To conduct an experimental study investigating the differences in the length of carriage of resistant bacteria, after exposure to a single course of antibiotics.	70	41	40	35	39	37	43.6	0.353
**327**	To develop digital thermometers for use with medicine fridges and freezers.	66	42	23	59	30	40	43.2	0.413
**328**	To identify ways to ensure that the systemic problems (and failings) of medication safety amongst healthcare professionals will not conflict with the current trend of increasing patient knowledge and awareness.	45	38	47	43	43	40	42.8	0.341

**329**	To investigate the effect on patient safety if medication is infused through central versus peripheral veins.	58	42	26	52	44	27	41.4	0.433
**330**	To research into producing a medicines handbook that classifies medication by disease and patient group, that can be applied to different geographic country contexts.	44	36	43	40	36	45	40.6	0.369
**331**	To conduct an exploratory study on the conditions and regulations needed to adopt the prescription to OTC switch.	52	32	38	43	27	43	39.4	0.282
**332**	To create recommendations to accurately identify a patient which could be applied to different institutional contexts.	50	42	27	48	38	25	38.2	0.329
**333**	To investigate the change in the status of the medication to create reliable processes.	29	31	24	28	28	26	27.4	0.325

Supplementary Table 3: Final ranks of 333 proposed research questions based on the scores from 27 experts in medication safety who were scoring mainly with a high-resource context in mind, and who represent a subset of the 42 scorers. Specific scores, ranging from 0-100, are presented for each of the 6 priority-setting criteria: answerability, effectiveness, innovativeness, implementability, potential for burden reduction and equitability. Questions are ranked according to their overall research priority scores (RPS), which also has a maximum theoretical range of 0-100%. Average expert agreement, which can theoretically range from 25-100%, is also provided for each question.

RANK HIC	RESEARCH QUESTION	ANSWERABLE	EFFECTIVE	INNOVATIVE	IMPLEMENTABLE	BURDEN REDUCED	EQUITABLE	RPS	AEA
1	To compare the benefits of pictorial information in medication instructions to written instructions alone, in improving medication safety. To what extent, in what contexts and formats is pictorial information most beneficial?	97	84	91	87	81	95	89.1	0.580
2	To identify and develop globally applicable pictograms for selected high-risk medications which would convey the critically important safety information.	93	88	80	89	94	90	89.0	0.599
3	To investigate how technologies could be appropriately implemented and scaled in LMICs to better ensure that drugs are not spoiled, diverted, counterfeited, and that supply chain performance is optimized to avoid stock outs and drug shortages.	91	100	77	88	90	87	88.7	0.500
4	To assess how the incidence of harm due to prescribing errors can be reduced by different interventions in low- and middle-income countries.	95	97	71	75	100	87	87.4	0.568

5	To investigate the role of health communication strategies to support patients with limited language proficiency, health literacy and education in taking medications safely.	90	86	71	85	83	100	85.9	0.599
6	To develop a predictive algorithm to identify individuals who are at risk of serious medication-related harm.	82	92	86	74	96	78	84.7	0.722
7	To identify affordable and effective methods of improving medication literacy among patients in resource limited settings.	89	84	79	87	83	84	84.6	0.549
8	To identify and create recommendations for the most effective approach to decision support alerts in electronic prescribing systems, the optimum sensitivity and specificity and criteria which should be used to enable prescriber to receive alerts but not receive alert fatigue.	96	89	74	91	89	68	84.6	0.667
9	To create patient knowledge-building tools for medication safety with critical thinking to ensure they are usable for people with low level of literacy, in a reliable format and addressing the role of internet as an information source.	89	82	77	80	79	96	83.7	0.617
10	To develop and validate a complexity score (c-score) for patients in need for de-prescribing which would help the physicians or pharmacists identify the high-risk patients	93	81	76	88	86	79	83.7	0.580

	who might develop drug-drug interactions.								
11	To identify pictograms which could be used to teach patients about their medicines.	97	72	72	83	88	89	83.7	0.506
12	To quantify the impact of electronic systems and knowledge-based clinical decision support on reducing medication-related harm in LMICs, by implementing this technology to smart-phones.	98	84	91	83	78	68	83.6	0.537
13	To assess the prevalence, main factors responsible and the effective interventions for preventing severe avoidable medication related patient harm in resource-limited settings through pilot studies.	88	89	64	82	87	84	82.2	0.543
14	To develop and validate a complexity score (c-score) to identify the patients who are at risk of readmission in 30 days due to medication errors which could be used by pharmacists and physicians.	91	80	73	89	86	70	81.3	0.611
15	To trial a pilot intervention study of the barcode system for medication in LMIC.	92	87	67	71	88	84	81.2	0.451
16	To improve medication safety for in-patients, through the application of ergonomics and human factors in the organization of the medications	91	82	75	86	88	66	81.2	0.593

	flow: order, distribution, stocking, preparation and administration.								
17	To identify the most effective empowerment methods and tools for patients and their caregivers to speak up when they see the potential for medication-related harm, especially applicable to patients in LMICs, as often the most impacted individuals are poorer and less educated.	86	75	83	73	78	93	81.1	0.537
18	To assess the impact of increasing the amount of trained human resources to reduce medication errors in low- and middle-income countries.	89	81	74	69	88	79	80.1	0.512
19	To conduct a study investigating the types of medication-related harm that occur in transitions between hospitals and primary care settings in LMIC.	98	83	58	78	89	75	80.0	0.537
20	To assess how decision support systems can be integrated with patient data, the culture barriers to rational prescribing in resource poor countries and how electronic prescribing systems relate to traditional medicine.	85	87	79	70	83	76	79.9	0.519
21	To identify indicators of medication safety that have been utilised in low-resource settings. What is known about their validity, reliability, and	95	80	68	83	75	78	79.7	0.537

	feasibility, and what potential indicators should be introduced?								
22	To test the use of a patient decision support system in order to ensure patient safety within self-management of medications in chronic conditions, with the remote support of health professionals and group of peers (patient experts).	88	74	85	79	76	76	79.6	0.568
23	To identify the reliable easily measured indicators to assess medication safety both in a facility level and also in national level.	86	82	68	84	77	73	78.4	0.562
24	To develop a mobile technology tool for patient and clinician use, aiding appropriate polypharmacy in LMIC.	89	82	85	67	69	75	77.9	0.463
25	To research into the utilization of digital health services monitoring large population health records to surveil adverse drug reaction signals.	89	76	83	82	81	58	77.9	0.506
26	To investigate how to ensure patient safety for patients utilizing oral home-based chemotherapy administration: maximising patient education and monitoring systems.	90	83	73	82	74	63	77.3	0.506
27	To determine the prevalence and potential clinical outcomes of medication errors in (1) primary care settings, and (2)	90	78	78	68	73	78	77.3	0.494

	hospitals in low resource countries.								
28	To investigate the effects of different interventions before/during/after the hospital discharge to ensure patients know and understand the information on their medication.	89	82	59	80	82	73	77.3	0.556
29	To assess how in resource poor countries/regions, evidence-based medication management systems which cover from prescribing to administering drugs can be implemented, and what organizational and cultural requirements are needed to implement such systems?	84	79	69	67	79	83	77.0	0.451
30	To identify what national strategies and/or policies for medication safety across high-, middle-, and low-income countries exist. What gaps remain in identifying and implementing these prevention strategies/policies?	98	80	57	68	76	83	77.0	0.537
31	To implement national level controlling and monitoring of safety of herbal medications.	83	72	61	83	88	71	76.5	0.463
32	To develop and evaluate a pilot patient medication recording system based on a cloud platform, accessible to all health care providers in prescribing and reviewing a patient's medication.	90	83	74	73	78	62	76.5	0.525

33	To evaluate the effectiveness of the Medication Without Harm Challenge by using Interrupted Time series to assess the implemented policies to prevent medication errors by measuring the situation prior and after (e.g. 6 months before and after) the introduction of the implementation of the policy.	93	83	69	78	74	63	76.4	0.506
34	Can implementing interventions related to barriers to de-prescribing, in patients with multi-morbidity and polypharmacy, minimise morbidity, mortality, reduce financial burden, improve medication adherence leading to enhance patient safety?	77	81	70	80	85	65	76.4	0.617
35	To evaluate which tools can be most useful to measure medication safety and determine the impact of measures that are implemented over time.	81	81	64	83	79	69	76.3	0.531
36	To identify factors that influence patient decision-making about polypharmacy, to develop decision aids for patients to help them take part on the discussion of taking multiple medications.	79	74	82	73	71	79	76.1	0.463
37	To assess cost-effective methods to keep track of what medicines a patient has taken and is taking, investigate how to record and share patient medication histories across different health infrastructures	76	83	62	79	83	71	75.8	0.512

	in order to reduce medication errors leading to medication-related patient harm?								
38	To explore novel ways, we can utilise technology (applications and other automation) to reduce adverse medication-related events. What is the role for technology in assisting patient monitoring and correct use of medications?	93	74	69	81	79	60	75.8	0.537
39	To create a cost-effectiveness study to identify mechanisms that support safe prescribing for elderly, poly-medicated patients.	89	91	57	80	74	64	75.7	0.512
40	To study the feasibility, impact and scalability of decision support tools to assist clinicians in safe medication prescription practice.	91	84	50	75	83	70	75.5	0.549
41	To conduct a multisite analysis investigating hospital adverse events. What has been the impact of electronic medical record (EMR) systems, the different modes of EMR, and optimal approaches in design and delivery?	93	82	63	68	83	64	75.4	0.481
42	To identify and create new indicators and metrics for medication safety to measure better the impact of medication safety work.	80	81	75	79	74	64	75.4	0.506

43	To examine the impact of the Essential Medicines List in LMIC, to prevent and address inappropriate polypharmacy at transitions of care.	82	63	80	77	67	83	75.4	0.395
44	To evaluate the impact of medication reconciliation in preventing medication errors in low-income countries.	89	82	59	67	83	71	75.1	0.451
45	To develop and test a core set of medication safety indicators, applicable for different contexts and countries worldwide. The set of core indicators may differ according to income level of country.	87	74	65	74	70	80	75.0	0.580
46	To identify high-risk situations applicable globally that should be programmed in all information systems (EHRs, CPOE, e-prescribing).	88	74	67	71	75	75	74.9	0.506
47	To identify certain group of diseases and patient groups that correlate to higher chances of experiencing a medication-related error and would such greatly benefit from conducting routing medication reviews and medication reconciliation.	81	85	50	80	81	71	74.9	0.617
48	To assess the prevalence of medication errors among patients who self-administer their medication in home setting and assess different methods of patient education as an intervention.	82	75	80	75	73	61	74.2	0.543

49	To design and develop sustainable, childproof and tamperproof containers which would ensure there is no risk to take dose higher than intended (e.g. impossible to dispense more than 10 ml before realigning it).	83	79	68	80	74	61	74.0	0.506
50	To identify best strategies to avoid inappropriate self-medication with antibiotics.	90	90	50	76	70	66	73.7	0.500
51	To perform a mixed-methods study examining the role patients and families play in contributing to medication errors due to non-adherence or over-adherence, and how much of this is due to issues with regards to access or health literacy? What evidence-based interventions exist?	85	74	72	75	68	68	73.6	0.630
52	To evaluate the role of human factors, ergonomics and error-proof designing of health care facilities as a medication safety strategy.	91	76	74	80	70	50	73.5	0.512
53	To evaluate forms of optimizing drug prescribing in the elderly in collaboration of pharmacists and GPs.	86	86	43	83	78	67	73.5	0.494
54	To evaluate forms of optimizing drug prescribing in the elderly in collaboration of pharmacists and GPs.	83	81	46	83	83	65	73.5	0.580

55	To perform a multi-modal analysis utilising a mixed methods approach assessing human factors, to analyze the most effective medication administration practices to prevent patient harm.	84	80	72	70	76	58	73.4	0.463
56	To investigate the impact of addressing high alert medications on morbidity and mortality in two pilot sites, one in LMIC and one HIC.	74	70	61	80	82	74	73.4	0.543
57	To examine the most frequent cause of hospital admissions in polypharmacy patients. What strategies can be implemented to reduce the risk?	86	88	43	76	92	55	73.2	0.506
58	To identify which remediable human factors are the most important to provide safe prescription and administration of medicines?	91	86	61	79	74	48	73.2	0.525
59	To evaluate the economic impact of interventions targeted to reducing medication-related adverse events.	88	83	63	68	71	68	73.1	0.494
60	To conduct a cost-effective analysis on interventions to reduce avoidable drug-related hospital admissions.	74	86	55	80	83	60	72.9	0.519
61	To conduct a study amongst health practitioners identifying at the practitioner-level barriers to implementation of evidence-based health literacy practices for explaining medications to patients and their families.	83	70	61	78	68	78	72.8	0.488

62	To evaluate medication discrepancies, communication, documentation and the role of medication reconciliation across care transitions in preventing medication-related patient harm.	95	89	36	73	84	59	72.6	0.549
63	To conduct an intervention study on the impact of medication dispensing technology in preventing and/or reducing medication errors.	100	83	45	79	76	53	72.5	0.494
64	To conduct an observational study examining the handling of high-risk medications across the continuum of care, and to identify strategies to ensure its safe use.	79	77	61	77	78	60	72.2	0.568
65	To develop a pilot implementation tool kit to improve medical and health care safety processes, applicable for different contexts and countries worldwide.	74	71	73	70	70	75	72.1	0.500
66	To conduct a multisite study analyzing the transfer and communication of medication-related information across health care settings, and between patients and providers. What are the weak points, how can current practices be strengthened?	93	68	52	77	79	62	71.9	0.519
67	To investigate the impact, feasibility and scalability of patient education techniques to promote reconciliation and adherence delivered in a variety	80	78	70	66	70	67	71.9	0.506

	of ways including: pharmacy, clinic, telephone, smart phone application.								
68	To commence a policy research on improving the impact of post-market surveillance systems to optimize patient care, drug effectiveness, and market corrections (drug recalls, etc.), including an assessment how the post-market surveillance systems differ across all countries and how this affects to medication safety.	88	70	60	80	68	65	71.7	0.469
69	To create effective techniques in medication error disclosure between health care professionals and to the patient, including legal statutes that can promote transparency.	83	69	67	71	73	67	71.6	0.469
70	To create a comparative study, which would assess the effectiveness of standardized, patient carried basic medication list in a developing country compared to more involved and complex tools (e.g. Best possible medication history lists) used in developed countries.	78	73	66	65	71	78	71.6	0.457
71	To evaluate the reduction in medication errors at a local level after introducing a program which aims to increase the role of patients in medication safety, engage them in their care and increase patient counselling, in	83	77	64	75	68	62	71.4	0.488

	minimizing the incidence of medication-related harm.								
72	To increase awareness among regulators and pharmaceutical industry how look-alike containers, poor visibility of key information (e.g. Active pharmaceutical ingredient) can cause medication errors and assess if dispensing only the necessary dose (breaking the fixed package when needed) would increase rational use of medicines.	89	80	50	78	83	50	71.4	0.488
73	To conduct a longitudinal study on how often systematic medication reviews should be performed in nursing home residents and how medication prescribing should be adjusted as treatment goals shift form preventive/curative to palliative/comforting care.	95	76	62	71	69	55	71.4	0.481
74	To design a uniform global standard of labelling for OTC medications and create recommendations for good labelling practice, including use of simple language.	81	75	68	70	75	60	71.4	0.481
75	To evaluate the role of service design and technology in improving safety at transitions of care.	79	79	68	71	79	53	71.3	0.488

76	To design and implement an information campaign aimed at patients to increase patient empowerment in reducing medication-related harm by addressing: transparency and completeness of information to be shared with healthcare providers, storage of medication, medication adherence, medication shifts, possible adverse effects and adverse drug reaction reporting.	82	75	50	77	66	77	71.2	0.506
77	To assess which aspects of organizational culture and/or context can help to prevent medication-related patient harm.	88	74	63	74	66	63	71.2	0.451
78	To investigate how having an understanding about health literacy can contribute towards ways that practitioners give information to patients about their medications.	78	69	66	64	71	79	71.0	0.426
79	To perform a qualitative study on patient's perspective: what is their role in medication management during transitions of care and what do they think it could be? Identify strategies used to engage patients in medication management during transitions of care. What are the barriers and facilitators to these strategies, and to assess the effectiveness of the identified strategies?	84	74	64	83	64	57	70.9	0.500

80	To conduct exploratory research into how potential medication safety issues can be predicted in a timely manner, with the use of algorithms based on clinical data.	74	74	87	68	72	50	70.8	0.426
81	To identify the most frequent patient-caused medication errors that occur at home and assess what strategies could be implemented.	87	74	70	74	73	48	70.8	0.537
82	To identify which factors, contribute to administration errors where medication is not given in nursing homes and care homes.	89	69	50	79	75	61	70.6	0.426
83	To perform a cost-effective analysis on interventions to reduce preventable medication-related hospitalizations , and medication-related falls and injuries in older adults.	82	82	46	68	80	64	70.3	0.593
84	To design technology assisted medication administration to reduce medication errors e.g. two-tier level identification.	88	80	50	75	79	50	70.2	0.475
85	To identify and build a platform which would allow patients to access their up-to-date medication list in all times regardless of the physical location.	82	82	48	71	81	55	69.7	0.500
86	To investigate cost-effective, multidisciplinary practices for optimising patient pharmacotherapy, especially for patients with chronic	80	77	48	76	80	57	69.6	0.512

	conditions and multi-morbidity.								
87	To examine errors in chemotherapy dosage and/or administration, what organisational changes can be implemented to prevent them from occurring?	88	79	50	79	68	55	69.6	0.451
88	To perform a study on nurses administering medicines in long-term care settings, what is the prevalence and root cause of medication administration adverse events?	100	71	45	71	65	64	69.6	0.457
89	To investigate identification systems (correct patient, correct route) and functional designs of hospitals to facilitate safe administration of medicines to patients with cognitive impairment.	80	71	44	78	78	66	69.4	0.426
90	To conduct a study across institutions identifying system-level barriers to implementation of evidence-based health literacy practices.	87	68	58	66	64	72	69.2	0.432
91	To identify globally applicable list of questions patients should know about their medications.	83	74	43	73	65	78	69.2	0.481
92	To conduct a multisite study evaluating interventions that have been successful in optimising medication error reporting and learning systems.	85	72	61	76	64	57	69.2	0.500

93	To investigate the response to opioids in middle-income countries: is the rise of opioid misuse, abuse, dependence associated with the increased availability of extended-release opioids.	78	61	68	68	76	64	69.1	0.383
94	To conduct epidemiology studies of adverse drug events and medical errors in non-academic tertiary settings such as nursing homes, cancer centres, and community hospitals.	86	64	55	71	74	64	69.0	0.469
95	To investigate how medication-related harm can be measured in feasible way, which requires minimal resources, that can be adapted to existing practices and can be measured ongoing basis.	66	75	74	70	69	60	69.0	0.463
96	What is the impact of current prescribing and medication use patterns during pregnancy on adverse birth outcomes in LMIC?	60	70	45	58	103	79	69.0	0.420
97	To test through pilot sites which are most fruitful and powerful combinations of practices, processes, and tools to prevent avoidable medication-related harm.	75	75	53	73	78	61	68.9	0.432
98	To design a patient pathway based monitoring framework and supporting metrics for evaluation of safe prescribing and administration of medicines.	72	70	69	68	71	63	68.9	0.444

99	To assess the reporting and learning of medication error systems at global and regional level and their impact on system change.	88	76	56	63	64	67	68.9	0.512
100	To identify measures which would enable safer medication-taking practices at home in LMIC.	78	68	53	68	69	78	68.8	0.352
101	To identify effective communication strategies to ensure medication safety in transitions of care (incl. Appropriate medications and education on those medicines)?	89	83	40	73	75	52	68.7	0.494
102	To determine the extent of harm contributed by sub-standard, spurious, falsified and counterfeit medicines (SSFFC) to patient safety. How to reduce the prevalence and incidence of SSFFC medicines in LMIC for better health outcomes?	63	75	56	63	76	79	68.6	0.451
103	What are the most frequent causes of severe, avoidable medication-related harm in high-, middle-, and low-income countries? If this is not known, what steps need to be taken to build and/or strengthen surveillance systems to identify medication-related harm?	77	66	55	69	75	69	68.5	0.512
104	To investigate the correlations between patient education and engagement with adherence to medication, inappropriate prescriptions and adverse drug events; and to identify which	73	64	69	74	66	64	68.3	0.457

	education tools are effective and sustainable.								
105	To conduct a pilot study on elderly patients in a long-term care facility/nursing home, do routine medication reviews reduce the overuse of medications?	95	74	52	79	68	43	68.3	0.475
106	To identify globally applicable list of minimum questions health care professionals should ask their patients before dispensing or prescribing medications.	89	67	57	74	62	60	67.9	0.475
107	To apply global standards on packaging and labelling to have uniformed place and way how and where the best before date and strength of the medicine is placed in the product.	81	73	61	68	68	58	67.9	0.426
108	To investigate incidents of medication-related patient harm, what role and to what extent does technology and technical complexity contribute?	85	65	68	68	68	53	67.7	0.438
109	To investigate the role of pharmacist's intervention in reducing medication errors through prospective cohort study. In which ways can the role of pharmacists be expanded to be actively involved in the medication prescribing process.	90	75	30	75	83	53	67.5	0.488

110	To conduct exploratory research on emerging technologies to prevent medication events from occurring, available for use in the patient's home and/or long-term care settings.	76	68	66	63	75	58	67.4	0.432
111	To investigate what are the most effective and cost-effective interventions carried out at the individual and community-level, to help reduce inappropriate antimicrobial use and reduce antimicrobial resistance.	90	75	35	74	76	53	67.1	0.481
112	To assess the benefits technology can play in reducing medication errors caused by verbal orders, during transition of care and in engaging and educating patients on their medication.	88	68	67	60	78	43	67.0	0.438
113	To assess the human factors which affect the decision-making process during prescribing, to teach best practices to doctors.	80	70	70	66	64	52	67.0	0.444
114	To investigate what areas of medication safety patients can best empower patients as guardians of safety for their own medication. What strategies can be utilized to promote this role and how can it be implemented?	74	65	63	72	61	68	67.0	0.389

115	To assess how globally data on medication errors and medication related harm is recorded beyond pharmacovigilance and implement a global database which would collect data on medication errors and near misses that especially low- and middle-income countries could report to.	69	63	62	65	63	80	66.9	0.451
116	To examine global legislation for medication safety, what policies are fundamental for assuring and improving medication safety?	84	68	55	64	69	61	66.8	0.475
117	To identify the predictor factors for patient readmission to hospital for medication-related problems in patients with complex conditions.	71	81	54	69	71	54	66.7	0.512
118	To identify the best practices for outpatient medication reconciliation followed by creating a toolkit out of the best practices (incl. interventions aimed at patients/caregivers, staff, clinics, health systems, and communities).	81	62	55	71	71	60	66.7	0.475
119	To conduct intervention study which utilizes mixed methods approach to transform a unit/hospital with high prevalence of medication errors to a low prevalence.	74	74	57	69	74	52	66.7	0.451

120	To examine errors related to look-alike, sound-alike medications that occur at the point of care and identify methods to prevent them.	89	73	48	73	73	45	66.6	0.469
121	To identify and evaluate potential strategies into how parents and caregivers can be effectively supported with providing high-risk medications to children.	79	73	55	75	55	63	66.5	0.512
122	To examine the role of Artificial Intelligence in reducing medication-related patient harm from the perspective of multiple stakeholders including clinicians, patients, consumers and administrators.	76	66	79	61	68	50	66.5	0.407
123	To implement an information campaign which would aim to inform population in in a more conscientious way about traditional medicines and food supplements possible risks and interactions with their usual medication.	82	63	50	74	69	61	66.4	0.395
124	To assess critical points of the medication process for injectable medicines in high-stakes environment, such as ED, anaesthesia, critical care, and develop strategies for improvement.	79	79	46	75	71	48	66.3	0.512
125	To identify strategies to enhance patient communication processes on medication management in hospitals and across transitions of care.	81	67	55	73	70	52	66.2	0.420

126	To examine the barriers to medication reporting amongst health care professionals in LMIC and what can be done to overcome them.	85	65	64	63	57	63	66.2	0.475
127	To conduct a patient-centred study on the effectiveness of medication counselling on decreasing misuse and identifying errors.	81	68	60	71	61	55	65.8	0.407
128	To examine each step of the medication process, what is the impact of interruptions in different stages and what implements can be made for improvement?	76	71	48	76	71	53	65.8	0.414
129	To review current prescribing guidelines and develop strategies to reduce overprescribing, particularly for opioids.	82	77	34	77	74	50	65.7	0.506
130	To identify a set of best trigger tools to identify a declared or potential adverse effect with high risk medications.	77	73	61	70	65	48	65.5	0.519
131	To assess and identify the weak links in the medication safety process chain to consolidate the local systems and resolve the occurring difficulties and differences in practice.	75	82	44	70	64	58	65.5	0.414
132	To identify which human factors are either involved in or contribute to medication-related patient harm.	83	68	48	74	70	50	65.4	0.451

133	To assess the role of regulators, manufacturers and the pharmaceutical industry in reducing medication related harm.	73	68	58	60	68	67	65.3	0.432
134	To evaluate the use of mobile technology for patients in supporting safer medicines managements, including monitoring of health conditions, education and prompting adherence?	83	63	65	66	63	53	65.3	0.420
135	To conduct a study into patient-user strategies, such as e-health and patient information, as facilitators for safe medication use and medication adherence.	82	60	60	73	60	56	65.3	0.506
136	To design and evaluate effective interventions designed to improve the uptake of health literacy practices related to medication teaching.	69	61	71	59	56	75	65.2	0.358
137	To implement interventions and WHO recommendations that would ensure the correct dosage and safety of medicines for children.	67	70	36	69	74	74	65.2	0.512
138	To identify weak points in the design of medication and/or its packaging and develop changes which will help reduce mistakes in medication administration.	83	70	53	68	70	48	65.1	0.383
139	To investigate the prevalence of adverse drug events and medication errors contributed by extemporaneous compounding medications for	65	70	66	61	66	63	65.1	0.494

	children, due to limited access to safe and effective medicines.								
140	To perform a study on the role of pharmacists in elderly patients transitions of care. What is their role, how can it be improved and what is the impact?	89	73	41	68	67	52	64.9	0.481
141	To develop standard protocols for the preparation, administration, monitoring of insulin in acute care settings, for application internationally.	72	74	39	70	73	61	64.7	0.500
142	To identify the impact of the availability of the Children`s Medicines List on the unmet needs of Children`s Medicines in Sub-Saharan Africa.	77	64	61	61	57	68	64.6	0.296
143	To perform an observational multisite study (across tertiary, long-term care, and primary care facilities) to assess how harm from drug interactions (including traditional and complementary medicines) are averted.	90	64	50	65	63	55	64.5	0.426
144	To investigate best practices to prevent adverse drug reactions for people with previous medication-related allergic reactions and hypersensitivities.	67	74	50	74	75	46	64.3	0.481
145	To assess the effectiveness of implementing a tool in detecting medication errors and harm in primary care?	69	70	48	73	63	64	64.3	0.438

146	To investigate methods of strengthening the review system of drug names, to prevent the confusion of look-alike, sound-alike medications.	80	70	45	71	73	48	64.3	0.438
147	To research into the development of novel electronic health record data entry methods, to facilitate accuracy and reduce errors.	69	61	60	68	71	55	64.0	0.426
148	To evaluate the implementation of QR coding for all medicines in their respective market and assess the public awareness of the need to verify the authenticity of medicines in countries which have implemented the QR coding.	80	62	63	63	63	53	63.7	0.420
149	To identify best strategies to inform the public that taking multiple medications can carry risks.	82	61	50	69	58	62	63.7	0.444
150	To identify and evaluate the primary national systems and/or strategies in place for identifying the burden and scope of medication-related harm in high-, middle-, and low-income countries?	86	55	48	60	65	69	63.6	0.451
151	To undertake exploratory research on the feasible implementation of a feedback system for physician prescribing practices.	75	67	60	73	67	40	63.5	0.444

152	To assess the effectiveness of different implementation strategies for sustaining and/or scaling up evidence-based interventions to reduce, eliminate or ameliorate medication-related patient harm?	70	70	48	68	68	58	63.4	0.401
153	To identify through literature review most effective interventions for medication safety and evaluate and measure their effectiveness through piloting sites using pre-set indicators.	84	71	36	73	68	48	63.4	0.463
154	To assess how innovative models of pharmaceutical care and diffusion of technology can be used to reduce medication related harm.	78	65	53	66	64	55	63.3	0.383
155	To investigate the problem of fake medicines and their international trade from surveillance, prevention, and interdiction standpoint to get a better and more resolute idea of the true scope and prevalence of substandard and falsified medicines.	60	68	55	58	70	69	63.2	0.407
156	To identify through systematic literature review best medication practices which could be implemented (modified to country context) in other countries?	78	63	40	65	71	63	63.2	0.426

157	To conduct a pilot study of the implementation of various strategies to reduce the use of potentially dangerous medications amongst elderly patients.	71	74	30	74	72	58	63.2	0.519
158	To establish the scale and burden of inappropriate non-evidence-based use of antipsychotic and antidepressants.	84	68	50	59	64	52	63.0	0.414
159	To assess the medication safety practices in long-term care facilities.	92	65	30	68	68	55	63.0	0.420
160	To assess the health outcomes in patients with chronic conditions who receive deliberate sensitization about possible side effects, adverse effects, interactions during their visits to the clinic against another group which receives the normal (no intervention) counselling.	79	58	58	64	64	55	63.0	0.389
161	To assess cost-effective ways to ensure medication compliance and investigate the role of mobile technology/assistive technology, to increase compliance in resource poor settings.	72	55	61	70	59	61	62.9	0.481
162	To investigate the reliability of medication safety indicators and tools in integrated care settings.	76	65	65	68	53	48	62.5	0.383

163	To conduct a multisite workplace analysis to determine required staffing levels to achieve minimal medication errors.	77	68	68	58	68	38	62.5	0.444
164	To identify methods to reduce inappropriate prescribing of antibiotics, and to develop strategies to minimize use.	76	69	33	78	70	48	62.3	0.475
165	To conduct a pilot intervention to develop a sharing platform among hospitals for describing errors and solutions.	77	68	26	68	64	69	62.2	0.469
166	To evaluate the current electronic prescribing process and pilot a redesign on indication and decision supporting drug of choice.	71	60	58	71	63	50	62.1	0.401
167	To perform a study evaluating the strategies to improve patient monitoring of adverse effects of medication.	83	64	45	64	63	52	62.1	0.401
168	To analyze the organizational and functional health care workforce: what can be better configured, developed, and managed to promote medication safety?	74	65	53	68	61	53	62.0	0.377
169	To develop safe self-administration models for patients or care takers with chronic diseases to self-administer their medication in hospital and home settings.	73	73	58	57	59	52	61.9	0.426

170	To identify gaps in deprescribing potentially inappropriate medicines in older people.	68	63	56	68	68	48	61.8	0.377
171	To create an evaluation framework that measures improvement initiatives so that those can be compared (by how effective, efficient, timely, patient-centred, equitable and safe each intervention is).	71	65	58	65	56	55	61.6	0.395
172	To reduce harm related to interactions by supporting creation of an app which would help prescribers in reducing the risk of interactions, supporting in decision making, offering possible alternative options, such as deprescribing, while considering patient's pathologies.	76	67	55	63	65	43	61.4	0.420
173	To perform an intervention study evaluating whether the provision on information about patient therapy to community pharmacists leads to a decrease in medication errors.	79	64	50	60	61	55	61.4	0.432
174	To conduct and evaluate the impact of medication reconciliation practices during transitions of care (e.g. post-hospital discharge).	86	64	20	64	82	52	61.4	0.519
175	To build methodological capacity in low- and medium-income countries (LMIC) with the aim of improving pharmaco-epidemiological research practices.	81	53	59	53	58	64	61.3	0.364

176	To assess how different interventions (including monitoring systems) reduce incidence of harm due to drug-drug interaction in poly-medicated elderly patients.	75	68	47	68	65	45	61.2	0.389
177	To identify through pilot studies best practices on how to make health care facilities and health care providers accept the existing essential drug lists; and accept the use of Standard Treatment Guidelines in countries where they exist and create them where they do not exist to ensure safe, rational use of medicines.	67	65	31	71	62	72	61.2	0.401
178	How to adapt patient safety indicators obtained from routinely collected data to different contexts and countries worldwide?	60	55	61	63	61	68	61.1	0.389
179	To apply modelling techniques on the intervention of specific medication problems in specific regions/countries, to ensure an effective and efficient allocation of resources.	61	58	58	51	61	78	60.9	0.358
180	To perform a cross-sectional study across primary care facilities assessing medication safety and exploring strategies in which it can be improved.	79	62	37	76	64	48	60.9	0.451
181	To perform a scoping review on devices/aids available to patients for reducing medication errors.	77	63	56	65	57	48	60.8	0.426

182	To perform a cost-effective analysis on interventions to improve medication adherence to evidence-based cardiovascular pharmacotherapy and other chronic conditions.	80	69	36	70	55	55	60.7	0.481
183	To study the feasibility, impact and scalability of awareness among patients and clinicians, that low kidney function as a major risk factor for medication-related patient safety events, delivered in a variety of ways - pharmacy, clinic, telephone, smart phone application?	68	58	54	67	63	52	60.6	0.420
184	To conduct a study within long-term care/nursing home facilities, examining the surveillance and reporting systems of adverse drug events and patient harm due to atypical antipsychotics.	84	61	43	60	63	52	60.5	0.438
185	To form and divulge basic notions about antibiotics, their uses, operations and sequential effects for patients to understand in what moments it is correct to prescribe them and the importance of following the guidelines recommended by their physician.	81	67	26	65	65	58	60.2	0.438
186	To identify the special considerations of medication safety in emergencies and create key policy recommendations on reduction of medication-related harm within the overall cycle of crises management.	71	60	64	60	58	48	60.1	0.383

187	To investigate the global burden of opioid over-doses and related deaths.	97	64	37	39	59	64	60.0	0.401
188	To collect evidence on the impact of patient empowerment, engagement and patient charters on reducing severe, avoidable medication related harm.	63	64	57	65	55	55	59.8	0.377
189	To identify cost-effective, sustainable methods for community-based promotion of medication safety interventions.	68	69	57	60	60	43	59.5	0.389
190	To critically evaluate procedures in the transfer of medication process to ensure safe inter-unit transfer.	74	69	33	58	68	55	59.4	0.432
191	To assess how to design the best environment for learning from medication-related incidents .	63	59	61	61	61	50	59.3	0.401
192	To investigate the relationship between the increased availability of prescription opioids and increased illicit drug use.	87	55	50	50	61	53	59.2	0.352
193	To identify the most effective strategies to enhance patient and caregiver`s engagement in medication safety.	64	61	43	69	68	50	59.2	0.414
194	To identify the best and most practical ways to measure preventable medication errors in real time and in patient-centered manner?	59	71	62	58	58	48	59.2	0.401

195	To identify methods of monitoring patient medication adherence and develop evidence-based practices to improve adherence, reduce patient harm and improve outcomes.	75	72	33	69	60	42	58.6	0.525
196	To evaluate effectiveness of treatments used to manage extravasation of intravenous medications to minimize medication related harm.	73	73	43	66	59	39	58.6	0.457
197	To design supporting tools for health care professionals which help to prescribe and dispense appropriate medications for old people.	54	59	41	70	69	57	58.5	0.426
198	To assess practices which can increase or decrease errors in anticoagulant therapy of patients who have transitions of care between secondary and primary care.	71	70	34	55	63	58	58.5	0.420
199	To investigate the change in medication safety practice after presenting medication error root cause analysis to healthcare professionals.	77	61	50	59	55	48	58.4	0.420
200	To assess the global prevalence, burden and geographical variation of serious medication related harm through a systematic literature review.	84	55	43	60	50	59	58.4	0.407
201	To investigate the impact of changing the package on the uptake of safe medication practices.	74	53	63	58	56	47	58.3	0.321

202	To investigate medication review process, which components are vital during medication review to ensure appropriate prescribing for patients with multiple morbidities.	61	64	43	62	69	50	58.2	0.432
203	To design a patient pathway based monitoring framework and supporting metrics for evaluation of safe prescribing and administration of medicines.	66	63	50	58	55	58	58.2	0.352
204	To create guidelines for safer use of paracetamol (acetaminophen) in children to address safe prescribing, safe and clear dosing schedule and safe dispensing and administration.	79	66	35	70	50	48	58.1	0.543
205	To identify the challenges and opportunities for health care professionals and the health care system to improve medication safety in transitions of care.	70	57	41	64	60	57	58.1	0.407
206	To investigate what percentage of medication errors are due to similarities in medication appearance.	71	63	45	64	62	43	58.0	0.438
207	To assess the prevalence of drug-drug interactions where one of the interacting medicines is prescription medicine and the other is non-prescription medicine.	79	60	43	64	48	55	57.9	0.383

208	To conduct an observational study in hospitals that systematically conducts medication reconciliation at discharge. How many adverse medication-related events, re-hospitalizations and deaths are avoided?	80	68	29	55	70	45	57.8	0.469
209	To implement data analytics to reduce medication related patient harm.	58	65	60	69	59	35	57.8	0.352
210	To investigate how to increase public reporting of health care associated infections and increase rewarding/giving incentives to healthcare facilities which meet quality and safety standards.	80	53	45	53	58	58	57.6	0.364
211	To examine how the role of health care professionals can be strengthened to ensure appropriate polypharmacy in the elderly.	64	55	43	62	64	57	57.5	0.395
212	To conduct a study investigating the impact of procurement based on clinical efficacy and safety, with the use of longitudinal data analytics thereby optimising benefits and minimising harm.	61	64	57	58	58	47	57.5	0.321
213	To identify and assess the best ways and tools for health care professionals to provide effective information about safe use of medications to patients and caregivers.	67	67	39	69	57	45	57.4	0.401

214	To perform a study investigating the underlying causes, extent, and the consequences of a lack of communication between different healthcare professionals and between healthcare professionals and their patients.	77	61	50	52	53	50	57.3	0.383
215	To identify collaborative team practices utilised by health care professionals to ensure appropriate drug monitoring and patient involvement?	70	59	52	57	65	38	57.0	0.395
216	To conduct an assessment on compliance and the reduced risk of adverse drug events in patients taking novel oral anticoagulants in lieu of warfarin.	72	63	34	70	59	43	56.9	0.475
217	To conduct an intervention study to determine whether handling of intravenous fluids alike medications (with protected access and patient-specific details) will reduce medication-related harm.	76	62	36	62	57	48	56.9	0.401
218	To ascertain and develop the role of clinical informaticians to bridge the gap between health IT design implementation and healthcare professionals.	69	52	63	60	53	45	56.9	0.395
219	To conduct a study investigating patient stock-piling of medications and conditions of their storage, their beliefs related to use of medicines and sources of medication information.	75	45	58	55	53	53	56.3	0.377

220	To develop and validate models focused on aspects of hospital layout and healthcare worker/patient flow to reduce HAIs.	71	47	53	61	59	47	56.3	0.340
221	To evaluate which strategies work best to ensure the accuracy of a patients` medication management across the continuum of care.	65	64	31	60	68	50	56.2	0.414
222	To assess how health care professionals can prevent drug interactions (drug-drug, drug-disease) in a simple and applicable way during daily working.	73	64	21	64	62	52	56.2	0.457
223	To identify strategies to improve compliance of medication in patients, caregivers and health professionals. What are effective newly developed tools for medication safety?	63	48	50	58	67	50	56.2	0.432
224	To identify the best and most cost-effective ways to transfer medication records in transitions of care.	73	68	39	55	64	38	56.1	0.420
225	What strategies can be implemented to reduce the overuse of medications with poor effectiveness in terminally ill cancer patients?	67	61	47	65	44	53	56.1	0.370
226	To investigate what are the specific barriers to address appropriate polypharmacy through interprofessional working in low-, middle- and high-income countries.	64	55	50	47	58	60	55.8	0.352

227	To examine whether the involvement of Health IT is functioning as expected to improve medication safety.	75	61	50	50	53	45	55.6	0.352
228	To investigate patient access and use of online medication record systems (such as OpenNotes) and medication schedules.	78	55	62	53	50	36	55.4	0.377
229	To conduct exploratory research on new methods to improve user-centred design and technology.	66	47	63	61	53	42	55.4	0.364
230	To explore ways to increase multi-professional team work and build respectful environment where teams recognize and respect the skills of each member in ensuring medication safety.	70	52	61	57	58	33	55.1	0.389
231	To assess the required global standards required to be in place before introducing a new digital system to any market.	68	53	56	62	47	44	55.0	0.340
232	To raise awareness amongst Ministries of Health and healthcare leaders in developing countries to highlight the importance of medication safety in primary care.	68	50	45	53	53	61	54.8	0.346
233	To identify medications that requires invasive laboratory monitoring (e.g. blood tests, ECG) and validating a standard recommendation.	76	52	38	69	62	31	54.7	0.444

234	To determine the incidence of antibiotics obtained without prescription or from earlier consultation by a physician, and how information technologies can reduce risky behaviours in LMIC.	71	53	50	50	59	44	54.6	0.333
235	To investigate the prescribing indication for opioids in middle- or third-world countries, has liberal prescribing for end-of-life care transitioned into use for non-cancer pain management.	74	50	53	41	57	53	54.6	0.284
236	To examine the impact of using external, non-biased safety testing companies, in evaluating the risks associated with potential brand names.	71	53	56	58	50	39	54.5	0.352
237	To investigate strategies to enhance patient engagement and better use patient information, to be applicable and achievable for patients across all income-level countries.	53	53	44	53	58	66	54.5	0.340
238	To assess patient attitudes and behaviours towards use and storage of medications in relation to health outcomes.	82	42	47	63	47	45	54.4	0.383
239	To identify and examine different types of surveillance programmes utilised to monitor medications with the potential for abuse.	70	47	58	61	50	39	54.2	0.346

240	To perform a qualitative cross-professional study that addresses how to optimise the role of the pharmacist in medical teams, medication administration and delivery.	77	55	40	55	55	43	54.2	0.395
241	To create a study assessing long-term adherence and compliance of patients who have complex multi-medication management, by looking from both the perspective of the patient and health care professional.	75	58	36	62	48	46	54.1	0.494
242	To examine ambulatory practice prescribing, which types of medications, or combinations of medications, have a high risk of safety problems in patients.	65	61	33	61	57	48	54.1	0.457
243	To create instructions for countries and institutions to help them to set up a good interdisciplinary concertation/coordination to avoid drug related errors/events.	69	57	45	50	53	50	54.0	0.377
244	To assess the lack of communication in transitions of care (i.e. at hospital discharge) by conducting an observational and interventional study?	77	57	26	61	61	41	54.0	0.457
245	To identify what strategies practitioners can apply individually to reduce medication errors.	78	60	43	60	45	38	53.8	0.401

246	To identify strategies to increase adverse drug event reporting/post marketing surveillance amongst physicians.	66	63	30	62	56	46	53.8	0.463
247	To assess improvements in medication safety after introducing restrictions on drug dispensing (requirement for a valid prescription).	74	55	45	53	53	43	53.6	0.370
248	To conduct an observational study to identify critical laboratory tests, which if incorrect, may lead to a higher risk of medication errors occurring.	63	61	47	50	50	50	53.5	0.333
249	To assess the influence of social determinants of health on adherence to medicines among patients with chronic diseases.	64	52	33	55	41	76	53.5	0.444
250	To evaluate the prevalence of unnecessary medications and food supplements, drug-drug interactions and drug-disease interactions among patients who take multiple medications.	70	63	29	53	64	42	53.3	0.370
251	To assess the nature and frequency of off-label medication use contributing to medication-related harm.	77	61	53	43	43	43	53.3	0.407
252	To assess the effectiveness of annual meetings with community pharmacist, to discuss which medicines (incl. OTC medications) the patient is taking and how and when they are taking them, can reduce medication-related harm	77	50	48	50	52	40	53.0	0.401

	among patients who take multiple medications.								
253	To examine incidents of product contamination and drug mislabelling. What techniques can be utilised to improve identification?	73	55	40	55	50	44	52.9	0.358
254	To evaluate if there is a difference in factors which contribute to medication errors depending on the level of health care (primary, secondary, tertiary) and health care system.	80	48	48	45	48	48	52.8	0.401
255	To conduct qualitative research on administrators and other managerial decision-makers addressing accountability on the decisions they make that create medication-related harm.	70	50	60	47	44	45	52.8	0.364
256	To critically evaluate implementation of a standardized opioid tolerance assessment and documentation prior to administration.	75	50	50	61	53	28	52.7	0.377
257	To investigate and develop model success stories utilising health IT.	68	47	50	53	50	47	52.5	0.333
258	To evaluate whether expert patient engagement in health systems can reduce severe, avoidable medication-related patient harm.	64	47	50	61	44	48	52.4	0.309

259	To assess various human factors, such as level of information on medications, as variates to measure which human factors in patients and health care professionals effect prevalence of medication errors.	52	47	50	55	62	47	52.3	0.302
260	To evaluate incidents of inappropriate polypharmacy and develop strategies to reduce harm and improve patient health outcomes.	62	60	29	55	63	45	52.3	0.389
261	To perform a study targeting health care prescribers, addressing how to promote effective communication and collaboration amongst other prescribing providers.	64	53	40	55	55	45	52.0	0.358
262	How does operationalization of 'resilience' in the health care system affect medication safety?	68	47	74	45	47	30	51.8	0.395
263	To conduct an intervention study enhancing patient involvement with their prescriber, does this empower patients to raise medication concerns promptly?	73	53	45	61	45	35	51.7	0.377
264	To conduct research into the development of expert systems encompassing a wide scope of patient information (including age, gender, genetic makeup, laboratory tests), to aid as a clinical decision support.	50	53	50	50	60	48	51.7	0.321

265	To conduct a study examining the role of individual health professionals and inter-professional collaboration, who is accountable for ensuring medication safety?	83	55	45	45	38	45	51.7	0.389
266	To determine factors that drive spread of HAIs and investigate new approaches that minimize the role of the healthcare environment in the spread of germs.	68	61	39	53	50	39	51.7	0.352
267	To investigate which quality improvement factors also reduce medication-related harm.	55	56	44	55	56	44	51.7	0.284
268	To identify the role of different actors in health care in reducing global incidence of medication related adverse events.	71	50	48	45	47	48	51.5	0.358
269	To develop and evaluate a system that monitors for the incompatibility of chemotherapy drugs with medications prescribed for other comorbidities, to reduce unwanted synergies.	73	58	26	57	60	35	51.4	0.426
270	To assess the efficacy of antibiotic delivery by the per lingual route, what is the impact on the gut flora and contribution to antimicrobial resistance?	74	53	53	44	47	38	51.4	0.333
271	To perform a study identifying medications (and/or combination of medications), that can potentially lead to acute	67	57	23	59	57	46	51.4	0.457

	kidney injury in high-risk critically ill patients.								
272	To conduct a multi-professional study implementing different interventions to investigate the effect on reducing medication errors.	60	66	37	50	55	40	51.3	0.383
273	To conduct a study on developing valid and reliable measures of patient engagement.	62	55	50	55	37	45	50.7	0.395
274	To assess the clinical significance of drug interactions based on recent studies and creating evidence-based strategies in deprescribing.	68	45	32	57	50	50	50.4	0.364
275	To create a study comparing effectiveness of two systems, one where medication errors and adverse drug reactions are reported to different entity/portal, and second where these two are combined and the approach is integrated.	75	48	63	47	36	33	50.3	0.401
276	To conduct a study on enhancing the role of pharmacists in multidisciplinary collaboration with prescribers. What are the attitudes of pharmacists and prescribers, how will collaboration best be utilised?	75	55	33	48	52	38	50.2	0.401

277	To measure which is the most sensitive moment, weekday and time of the day for occurrence of medication errors: ordering, prescribing, preparing, dispensing, administering or monitoring?	70	52	36	50	52	39	49.9	0.383
278	To identify the challenges involving the patient and their caregivers in the campaign to improve medication safety, and what resources and support is required?	58	50	39	53	53	48	49.9	0.309
279	To conduct a qualitative study for patients, what are their priorities for reducing the risk of medication-related harm?	67	45	45	60	38	43	49.6	0.389
280	To investigate the impact of personalised medicines.	55	48	53	47	45	50	49.5	0.340
281	To assess through a pilot study if each time patient interacts with health care system the health care professionals would be obligated to look at least the past three visits back would reduce medication-related harm and help health care professionals to recognize medication-related harm	67	47	58	42	47	36	49.5	0.389
282	To address the key issues in reconciling prescriptions and administration across settings through a pilot study.	75	50	18	60	53	40	49.3	0.420

283	To conduct exploratory research expanding the role of nurses to take on leadership roles in preventing medication-related patient harm.	61	47	42	56	47	39	48.5	0.315
284	To conduct a multisite/multimodal study evaluating the role and impact of utilising digital methods in the health care process to reduce medication errors.	63	48	38	55	47	40	48.3	0.340
285	To conduct a qualitative study seeking to examine how to enhance patient engagement amongst patients and their family, to improve medication safety.	74	45	29	53	37	53	48.3	0.370
286	To evaluate the efficacy of generic antibiotics compared to their original patented brand. Do they have the same impact on antibiotic-resistant bacteria in the digestive flora?	71	44	44	42	47	41	48.2	0.340
287	To systematically evaluate the most appropriate devices to deliver intravenous medications assessing the risk of complications, and to additionally investigate.	58	55	38	52	53	33	47.9	0.389
288	To gather evidence about the clinically important role of pharmacists in medication safety.	66	52	24	50	55	40	47.9	0.420
289	To perform a qualitative study investigating what are the perceived causes of unsafe medication practices.	79	45	42	48	40	33	47.8	0.444

290	To assess the magnitude of severe drug interactions due to poly-pharmacy in elderly.	64	53	31	50	50	37	47.4	0.389
291	To conduct a study designed to investigate the intentional non-adherence of medication.	65	45	45	39	43	46	47.4	0.407
292	To assess nurses' skills to do dose calculations after absence from work and compare the drug calculation skills of recent nursing students to experienced nurses who have just came back to work from leave which length has been predetermined to be significant enough (e.g. in maternity leave).	83	37	42	47	47	29	47.4	0.401
293	To conduct a study assessing prescribing of non-approved medication and off-label use; and to investigate methods to reduce.	61	57	45	41	40	40	47.4	0.401
294	To develop Shared Care Guidelines for selected medicines, to promote safe continuity of care in the community.	58	47	29	47	45	55	46.9	0.370
295	To assess in institutional level the obstacles and facilitators of adverse drug event reporting to increase the reporting	85	44	36	50	34	30	46.7	0.457
296	To identify the enablers and barriers to the routine clinical recognition, diagnosis and recording of serious medication-related harm.	67	45	48	45	44	32	46.6	0.352

297	To assess through an observational study how adverse drug events happen and implement interventions to prevent them from occurring.	56	54	29	54	54	32	46.5	0.475
298	To conduct an intervention study in hospital emergency departments evaluating the effectiveness of strategies (organizational/functional) to reduce delay and overcrowding.	66	47	32	42	39	50	46.2	0.352
299	To assess the risk of polypharmacy for individual patients and develop cost-effective solutions.	55	61	31	39	56	34	45.9	0.370
300	To identify the most frequent diagnostic errors and implement practices to prevent them.	53	56	39	41	47	39	45.8	0.358
301	To conduct exploratory research on how pharmaceutical professionals can adopt medication safety as their principal core societal role.	63	41	44	42	41	39	45.1	0.346
302	To compare generic marking of every individual medication and dosage against existing medication in improving medication safety?	63	47	40	40	39	41	44.8	0.296
303	To assess the occurrence rate, patterns and trends of medication errors in communities across countries.	66	43	31	38	37	53	44.4	0.401

304	To perform an observational study to identify which laboratory tests can early diagnose a medication error.	45	42	50	39	39	47	43.8	0.352
305	Assessing the benefits on patient safety and efficacy of marking expiration month and date on tablets.	63	36	44	45	35	36	43.3	0.370
306	To conduct an analysis on what hinders the voluntary reporting of medication errors even when guidelines exist.	74	41	28	48	36	32	43.2	0.469
307	To perform a qualitative study within the health care workforce focusing on what style of leader health care professionals desire to prevent avoidable medication-related harm?	66	40	47	31	40	33	43.0	0.426
308	To identify methods to ensure that each patient is taking the correct medication	45	50	19	45	55	42	42.8	0.340
309	To assess the role of nurses in preventing drug-drug interactions.	66	39	33	43	43	33	42.7	0.432
310	To identify through pilot studies ways to prevent inappropriate use and over-prescribing due to financial incentives to physicians.	53	40	48	42	33	40	42.5	0.383
311	To conduct a study identifying what measures are required to develop the health care team`s understanding towards the	58	42	36	42	39	38	42.5	0.352

	Standard Protocol of Medicine Reconciliation.								
312	To perform a qualitative study on perceptions of community pharmacists towards the evaluation of knowledge and skills related to safe medication practice.	80	33	29	36	39	32	41.7	0.407
313	To conduct a study investigating clinical situations that lie outside the guidelines, is there an increased incidence of unnecessary bridging with heparin or low molecular weight heparin?	57	41	36	55	36	25	41.5	0.426
314	To conduct a longitudinal observational study of patient medication non-adherence on health outcomes.	62	42	35	39	31	40	41.5	0.494
315	To assess the benefits and risks of medications affecting the central nervous system, such as antipsychotics, antidepressants, anticonvulsants, benzodiazepines, in patients with complex conditions such as ADHD, substance abuse/misuse, and chronic pain.	52	52	17	33	50	43	41.3	0.438
316	To perform qualitative ethnographic research exploring health care staff perspectives on safety and the role they play.	62	38	47	40	26	34	41.2	0.407

317	To create recommendations to accurately identify a patient which could be applied to different institutional contexts.	56	43	25	56	43	23	41.2	0.340
318	To identify which elements globally in robust health systems, help to avert medication related harm.	57	43	30	38	39	40	41.2	0.407
319	To compare the efficacy of generic medication to the original index drug and all other generic forms?	55	36	31	39	36	47	40.8	0.370
320	To create up-to-date recommendations for safe and good prescribing practices.	41	54	20	43	45	36	40.1	0.438
321	To identify ways to ensure that the systemic problems (and failings) of medication safety amongst healthcare professionals will not conflict with the current trend of increasing patient knowledge and awareness.	42	31	42	39	42	43	39.6	0.358
322	To perform a mixed method study examining the role of pharmacists and non-physicians in enhancing medication safety.	60	43	24	43	33	30	38.6	0.420
323	To conduct a study exploring implementation methods of drug classification systems in LMIC.	53	27	32	44	32	41	38.1	0.333

324	To assess the consequences to the individual's well-being and to their effectiveness when the workplace pursues complete elimination of avoidable harm.	45	42	38	37	39	26	38.0	0.395
325	To undertake an examination of health policy analyses in LMIC. Are the measures sufficient?	53	33	27	37	35	40	37.4	0.321
326	To develop digital thermometers for use with medicine fridges and freezers.	63	36	13	58	19	36	37.4	0.438
327	To investigate the effect on patient safety if medication is infused through central versus peripheral veins.	55	36	19	50	40	20	36.7	0.475
328	To develop clinical guidelines for rarely used drugs and perform audits on use.	50	36	44	29	28	29	36.1	0.364
329	To analyze and identify the root cause of multidrug resistance in the treatment process to create more effective interventions.	47	32	21	42	38	34	35.7	0.383
330	To research into producing a medicines handbook that classifies medication by disease and patient group, that can be applied to different geographic country contexts.	40	31	36	33	31	39	34.9	0.401
331	To conduct an experimental study investigating the differences in the length of carriage of resistant bacteria, after exposure to a single course of antibiotics.	70	29	22	22	24	21	31.3	0.432

332	To conduct an exploratory study on the conditions and regulations needed to adopt the prescription to OTC switch.	50	18	29	36	15	34	30.3	0.352
333	To investigate the change in the status of the medication to create reliable processes.	25	27	19	27	27	21	24.4	0.352

Supplementary Table 4: Final ranks of 333 proposed research questions based on the scores from 10 experts in medication safety who were scoring mainly with a LMIC context in mind, and who represent a subset of the 42 scorers. Specific scores, ranging from 0-100, are presented for each of the 6 priority-setting criteria: answerability, effectiveness, innovativeness, implementability, potential for burden reduction and equitability. Questions are ranked according to their overall research priority scores (RPS), which also has a maximum theoretical range of 0-100%. Average expert agreement, which can theoretically range from 25-100%, is also provided for each question.

RANK LMIC	RESEARCH QUESTION	ANSWERABLE	EFFECTIVE	INNOVATIVE	IMPLEMENTABLE	BURDEN REDUCED	EQUITABLE	RPS	AEA
1	To assess and identify the weak links in the medication safety process chain to consolidate the local systems and resolve the occurring difficulties and differences in practice.	88	100	94	94	94	100	94.8	0.733
2	To assess the prevalence, main factors responsible and the effective interventions for preventing severe avoidable medication related patient harm in resource-limited settings through pilot studies.	94	94	100	89	94	94	94.3	0.750
3	To investigate the impact of addressing high alert medications on morbidity and mortality in two pilot sites, one in LMIC and one HIC.	100	100	88	94	89	89	93.3	0.800
4	To identify what national strategies and/or policies for medication safety across high-, middle-, and low-income countries exist. What gaps remain in identifying and implementing these prevention strategies/policies?	94	100	75	94	100	94	92.7	0.717
5	To identify the most effective empowerment methods and tools for patients and their caregivers to speak up when they see the potential for medication-related harm, especially applicable to patients in LMICs, as often the	89	90	90	95	94	95	92.2	0.833

	most impacted individuals are poorer and less educated.								
6	What are the most frequent causes of severe, avoidable medication-related harm in high-, middle-, and low-income countries? If this is not known, what steps need to be taken to build and/or strengthen surveillance systems to identify medication-related harm?	94	100	75	94	94	94	92.0	0.750
7	To identify and create new indicators and metrics for medication safety to measure better the impact of medication safety work.	93	93	93	93	93	86	91.7	0.583
8	To assess the reporting and learning of medication error systems at global and regional level and their impact on system change	94	89	81	94	89	100	91.2	0.750
9	To evaluate the prevalence of unnecessary medications and food supplements, drug-drug interactions and drug-disease interactions among patients who take multiple medications.	94	100	69	94	94	94	91.1	0.767
10	To investigate the correlations between patient education and engagement with adherence to medication, inappropriate prescriptions and adverse drug events; and to identify which education tools are effective and sustainable.	94	95	80	100	95	80	90.7	0.850
11	To build methodological capacity in low- and medium-income countries (LMIC) with the aim of improving pharmaco-epidemiological research practices.	89	94	78	89	94	100	90.7	0.767

12	To examine the barriers to medication reporting amongst health care professionals in LMIC and what can be done to overcome them.	94	95	70	95	95	94	90.6	0.817
13	To develop safe self-administration models for patients or care takers with chronic diseases to self-administer their medication in hospital and home settings.	94	89	83	89	94	94	90.6	0.717
14	To assess the impact of increasing the amount of trained human resources to reduce medication errors in low- and middle-income countries	89	95	80	95	95	89	90.5	0.800
15	To identify the most frequent patient-caused medication errors that occur at home and assess what strategies could be implemented.	94	94	88	89	89	89	90.4	0.700
16	To conduct a patient-centred study on the effectiveness of medication counselling on decreasing misuse and identifying errors.	89	89	83	89	94	94	89.8	0.750
17	To develop and validate a complexity score (c-score) to identify the patients who are at risk of readmission in 30 days due to medication errors which could be used by pharmacists and physicians	94	94	94	89	89	78	89.8	0.750
18	To perform a multi-modal analysis utilising a mixed methods approach assessing human factors, to analyze the most effective medication administration practices to prevent patient harm.	92	88	79	93	88	100	89.7	0.567

19	To perform a qualitative study on patient's perspective: what is their role in medication management during transitions of care and what do they think it could be? Identify strategies used to engage patients in medication management during transitions of care. What are the barriers and facilitators to these strategies, and to assess the effectiveness of the identified strategies?	100	94	81	89	89	83	89.4	0.683
20	To investigate identification systems (correct patient, correct route) and functional designs of hospitals to facilitate safe administration of medicines to patients with cognitive impairment.	86	93	93	81	88	94	89.0	0.583
21	To conduct qualitative research on administrators and other managerial decision-makers addressing accountability on the decisions they make that create medication-related harm.	93	88	93	88	88	86	89.0	0.600
22	To identify strategies to enhance patient communication processes on medication management in hospitals and across transitions of care.	94	90	89	85	85	90	88.9	0.767
23	To investigate medication review process, which components are vital during medication review to ensure appropriate prescribing for patients with multiple morbidities.	100	90	89	90	85	78	88.6	0.800
24	To examine global legislation for medication safety, what policies are fundamental for assuring and improving medication safety?	94	94	69	94	94	88	88.5	0.650
25	To identify medications that requires invasive laboratory monitoring (e.g. blood tests, ECG) and validating a standard recommendation.	94	88	86	88	94	81	88.2	0.650

26	To conduct an observational study to identify critical laboratory tests, which if incorrect, may lead to a higher risk of medication errors occurring.	94	88	100	81	81	86	88.2	0.617
27	To conduct an intervention study enhancing patient involvement with their prescriber, does this empower patients to raise medication concerns promptly?	85	90	89	90	90	85	88.1	0.750
28	To assess how the incidence of harm due to prescribing errors can be reduced by different interventions in low- and middle-income countries.	89	90	70	95	90	94	88.1	0.767
29	To assess cost-effective methods to keep track of what medicines a patient has taken and is taking, investigate how to record and share patient medication histories across different health infrastructures in order to reduce medication errors leading to medication-related patient harm?	94	94	72	94	94	78	88.0	0.733
30	To identify the most frequent diagnostic errors and implement practices to prevent them.	94	100	56	94	94	89	88.0	0.733
31	To conduct a study investigating the types of medication-related harm that occur in transitions between hospitals and primary care settings in LMIC.	94	89	67	94	89	94	88.0	0.717
32	To improve medication safety for in-patients, through the application of ergonomics and human factors in the organization of the medications flow: order, distribution, stocking, preparation and administration.	92	93	83	81	86	93	87.9	0.533
33	To create patient knowledge-building tools for medication safety with critical thinking to ensure they are usable for people with low level of literacy, in a	94	94	89	89	89	72	87.8	0.733

	reliable format and addressing the role of internet as an information source.								
34	To assess the nature and frequency of off-label medication use contributing to medication-related harm.	88	94	63	94	94	94	87.5	0.633
35	To apply modelling techniques on the intervention of specific medication problems in specific regions/countries, to ensure an effective and efficient allocation of resources.	81	88	100	88	81	88	87.5	0.617
36	To investigate how to ensure patient safety for patients utilizing oral home-based chemotherapy administration: maximising patient education and monitoring systems.	94	88	79	94	78	93	87.5	0.650
37	To analyze the organizational and functional health care workforce: what can be better configured, developed, and managed to promote medication safety?	88	94	78	89	94	81	87.4	0.683
38	To identify affordable and effective methods of improving medication literacy among patients in resource limited settings	94	100	57	89	90	94	87.4	0.717
39	To examine the impact of the Essential Medicines List in LMIC, to prevent and address inappropriate polypharmacy at transitions of care.	89	94	75	89	89	88	87.3	0.717
40	To examine errors in chemotherapy dosage and/or administration, what organisational changes can be implemented to prevent them from occurring?	94	94	56	94	83	100	87.0	0.667

41	To assess how globally data on medication errors and medication related harm is recorded beyond pharmacovigilance and implement a global database which would collect data on medication errors and near misses that especially low- and middle-income countries could report to.	83	89	94	83	83	89	87.0	0.700
42	To determine the prevalence and potential clinical outcomes of medication errors in (1) primary care settings, and (2) hospitals in low resource countries.	90	95	67	90	90	90	86.9	0.767
43	To identify globally applicable list of minimum questions health care professionals should ask their patients before dispensing or prescribing medications.	89	83	83	89	83	94	86.9	0.717
44	To assess critical points of the medication process for injectable medicines in high-stakes environment, such as ED, anaesthesia, critical care, and develop strategies for improvement.	94	100	63	94	94	75	86.8	0.733
45	To identify the best and most cost-effective ways to transfer medication records in transitions of care	88	89	83	89	89	83	86.8	0.700
46	To conduct a study assessing prescribing of non-approved medication and off-label use; and to investigate methods to reduce.	89	94	69	81	94	94	86.7	0.617
47	To conduct a pilot study of the implementation of various strategies to reduce the use of potentially dangerous medications amongst elderly patients.	94	94	75	83	83	89	86.6	0.717
48	To evaluate the reduction in medication errors at a local level after introducing a program which aims to increase the role of patients in medication safety,	83	94	75	89	94	83	86.6	0.700

	engage them in their care and increase patient counselling, in minimizing the incidence of medication-related harm.								
49	To implement interventions and WHO recommendations that would ensure the correct dosage and safety of medicines for children.	100	94	63	88	88	88	86.5	0.617
50	To perform a scoping review on devices/aids available to patients for reducing medication errors.	94	88	81	88	88	81	86.5	0.617
51	To identify what strategies practitioners can apply individually to reduce medication errors.	88	89	69	89	89	94	86.1	0.650
52	To identify and assess the best ways and tools for health care professionals to provide effective information about safe use of medications to patients and caregivers.	89	95	60	90	90	90	85.6	0.750
53	To perform a qualitative cross-professional study that addresses how to optimise the role of the pharmacist in medical teams, medication administration and delivery.	89	89	72	88	89	88	85.6	0.683
54	To identify certain group of diseases and patient groups that correlate to higher chances of experiencing a medication-related error and would such greatly benefit from conducting routing medication reviews and medication reconciliation.	83	95	80	90	85	80	85.6	0.750
55	To identify the most effective strategies to enhance patient and caregiver`s engagement in medication safety.	83	90	65	90	90	95	85.6	0.767

56	To examine the most frequent cause of hospital admissions in polypharmacy patients. What strategies can be implemented to reduce the risk?	89	83	81	83	89	88	85.5	0.667
57	To conduct a study on developing valid and reliable measures of patient engagement.	90	90	63	90	90	90	85.4	0.733
58	To conduct a study across institutions identifying system-level barriers to implementation of evidence-based health literacy practices.	88	88	88	78	83	89	85.4	0.600
59	To develop a pilot implementation tool kit to improve medical and health care safety processes, applicable for different contexts and countries worldwide.	88	88	75	88	88	88	85.4	0.583
60	To identify and evaluate potential strategies into how parents and caregivers can be effectively supported with providing high-risk medications to children.	83	90	83	85	85	85	85.3	0.750
61	To develop a predictive algorithm to identify individuals who are at risk of serious medication-related harm.	100	83	94	83	83	67	85.2	0.683
62	To assess the occurrence rate, patterns and trends of medication errors in communities across countries.	94	89	56	89	89	94	85.2	0.667
63	To assess the magnitude of severe drug interactions due to poly-pharmacy in elderly.	89	94	61	89	89	89	85.2	0.683
64	To evaluate the role of service design and technology in improving safety at transitions of care	86	88	88	88	88	75	85.1	0.567
65	To assess the prevalence of medication errors among patients who self-administer their medication in home setting and assess different methods of	85	85	85	85	85	85	85.0	0.800

	patient education as an intervention.								
66	To design supporting tools for health care professionals which help to prescribe and dispense appropriate medications for old people	88	88	71	81	94	88	84.8	0.583
67	To assess the global prevalence, burden and geographical variation of serious medication related harm through a systematic literature review.	100	89	75	83	83	78	84.7	0.717
68	To undertake an examination of health policy analyses in LMIC, are the measures sufficient?	100	83	69	89	83	83	84.6	0.633
69	To identify the special considerations of medication safety in emergencies and create key policy recommendations on reduction of medication-related harm within the overall cycle of crises management.	86	88	71	81	94	88	84.5	0.550
70	To identify the enablers and barriers to the routine clinical recognition, diagnosis and recording of serious medication-related harm	100	88	58	88	88	86	84.4	0.567
71	To conduct an analysis on what hinders the voluntary reporting of medication errors even when guidelines exist.	81	88	81	88	88	81	84.4	0.600
72	To perform qualitative ethnographic research exploring health care staff perspectives on safety and the role they play.	100	81	81	88	81	75	84.4	0.567
73	To evaluate medication discrepancies, communication, documentation and the role of medication reconciliation across care transitions in preventing medication-related patient harm	86	89	67	89	94	81	84.3	0.667

74	To evaluate whether expert patient engagement in health systems can reduce severe, avoidable medication-related patient harm.	83	94	94	83	78	72	84.3	0.667
75	To identify collaborative team practices utilised by health care professionals to ensure appropriate drug monitoring and patient involvement?	88	89	69	83	83	94	84.3	0.617
76	To examine how the role of health care professionals can be strengthened to ensure appropriate polypharmacy in the elderly.	72	83	72	94	89	94	84.1	0.683
77	To develop and test a core set of medication safety indicators, applicable for different contexts and countries worldwide. The set of core indicators may differ according to income level of country.	86	88	81	81	81	88	84.1	0.550
78	To identify methods to reduce inappropriate prescribing of antibiotics, and to develop strategies to minimize use.	88	89	56	94	89	89	84.0	0.700
79	To perform an observational multisite study (across tertiary, long-term care, and primary care facilities) to assess how harm from drug interactions (including traditional and complementary medicines) are averted.	93	81	81	75	88	86	83.9	0.583
80	To evaluate which strategies work best to ensure the accuracy of a patients` medication management across the continuum of care.	81	89	72	89	89	83	83.9	0.667
81	To investigate strategies to enhance patient engagement and better use patient information, to be applicable and achievable for patients across all income-level countries.	75	83	83	89	83	89	83.8	0.617

82	To conduct an assessment on compliance and the reduced risk of adverse drug events in patients taking novel oral anticoagulants in lieu of warfarin.	100	81	71	100	79	71	83.8	0.583
83	To conduct exploratory research on how pharmaceutical professionals can adopt medication safety as their principal core societal role.	81	83	83	83	83	88	83.7	0.683
84	To determine the extent of harm contributed by sub-standard, spurious, falsified and counterfeit medicines (SSFFC) to patient safety. How to reduce the prevalence and incidence of SSFFC medicines in LMIC for better health outcomes?	69	88	63	94	94	94	83.3	0.600
85	To investigate cost-effective, multidisciplinary practices for optimising patient pharmacotherapy, especially for patients with chronic conditions and multi-morbidity.	83	90	67	90	90	80	83.3	0.767
86	To evaluate the impact of medication reconciliation in preventing medication errors in low-income countries.	100	85	55	75	85	100	83.3	0.767
87	To assess in institutional level the obstacles and facilitators of adverse drug event reporting to increase the reporting	89	83	61	94	83	89	83.3	0.633
88	To investigate how medication-related harm can be measured in feasible way, which requires minimal resources, that can be adapted to existing practices and can be measured ongoing basis.	75	88	81	88	81	88	83.3	0.567
89	To design a patient pathway based monitoring framework and supporting metrics for evaluation of safe prescribing and administration of medicines	80	90	90	70	90	80	83.3	0.350

90	To investigate what are the specific barriers to address appropriate polypharmacy through interprofessional working in low-, middle- and high-income countries	88	88	56	88	88	94	83.3	0.583
91	To create instructions for countries and institutions to help them to set up a good interdisciplinary concertation/coordination to avoid drug related errors/events.	75	83	75	89	83	94	83.2	0.600
92	To identify the best and most practical ways to measure preventable medication errors in real time and in patient-centered manner?	86	94	56	88	88	88	83.0	0.567
93	To examine errors related to look-alike, sound-alike medications that occur at the point of care and identify methods to prevent them.	93	88	50	88	88	93	83.0	0.550
94	To identify a set of best trigger tools to identify a declared or potential adverse effect with high risk medications.	86	88	93	79	79	75	83.0	0.483
95	To identify effective communication strategies to ensure medication safety in transitions of care (incl. Appropriate medications and education on those medicines)?	94	88	63	83	88	83	83.0	0.583
96	To design and implement an information campaign aimed at patients to increase patient empowerment in reducing medication-related harm by addressing: transparency and completeness of information to be shared with healthcare providers, storage of medication, medication adherence, medication shifts, possible adverse effects and adverse drug reaction reporting.	80	85	75	90	90	78	83.0	0.750

97	To identify cost-effective, sustainable methods for community-based promotion of medication safety interventions.	83	86	79	79	86	86	82.9	0.483
98	To examine incidents of product contamination and drug mislabeling. What techniques can be utilised to improve identification?	79	94	69	88	88	81	82.9	0.550
99	To assess the lack of communication in transitions of care (i.e. at hospital discharge) by conducting an observational and interventional study?	100	83	56	89	83	83	82.4	0.700
100	To develop and validate a complexity score (c-score) for patients in need for de-prescribing which would help the physicians or pharmacists identify the high-risk patients who might develop drug-drug interactions.	89	89	89	78	78	72	82.4	0.650
101	To identify methods to ensure that each patient is taking the correct medication	89	94	56	94	78	83	82.4	0.683
102	To perform a qualitative study investigating what are the perceived causes of unsafe medication practices.	94	89	56	89	83	83	82.4	0.650
103	To explore ways to increase multi-professional team work and build respectful environment where teams recognize and respect the skills of each member in ensuring medication safety	72	83	89	83	83	83	82.4	0.683
104	To identify best strategies to inform the public that taking multiple medications can carry risks.	89	83	61	89	83	89	82.4	0.667
105	To investigate the role of health communication strategies to support patients with limited language proficiency, health literacy and education in taking medications safely.	79	88	71	81	88	88	82.3	0.517

106	To assess how health care professionals can prevent drug interactions (drug-drug, drug-disease) in a simple and applicable way during daily working	88	83	63	94	78	88	82.2	0.650
107	To evaluate if there is a difference in factors which contribute to medication errors depending on the level of health care (primary, secondary, tertiary) and health care system	81	83	72	89	83	83	82.1	0.650
108	To evaluate forms of optimizing drug prescribing in the elderly in collaboration of pharmacists and GPs.	89	88	56	89	83	88	81.9	0.650
109	To conduct a study identifying what measures are required to develop the health care team`s understanding towards the Standard Protocol of Medicine Reconciliation.	88	83	63	88	83	88	81.9	0.567
110	To investigate methods of strengthening the review system of drug names, to prevent the confusion of look-alike, sound-alike medications.	93	86	50	88	88	88	81.8	0.533
111	To investigate what areas of medication safety patients can best empower patients as guardians of safety for their own medication. What strategies can be utilized to promote this role and how can it be implemented?	81	83	72	83	83	88	81.8	0.567
112	To evaluate the role of human factors, ergonomics and error-proof designing of health care facilities as a medication safety strategy	81	83	81	83	83	78	81.7	0.583
113	To assess which aspects of organizational culture and/or context can help to prevent medication-related patient harm	79	83	69	83	83	93	81.7	0.617
114	To identify which elements globally in robust health systems,	81	88	71	88	88	75	81.7	0.533

	help to avert medication related harm								
115	To collect evidence on the impact of patient empowerment, engagement and patient charters on reducing severe, avoidable medication related harm.	85	85	75	95	75	75	81.7	0.700
116	To perform a study investigating the underlying causes, extent, and the consequences of a lack of communication between different healthcare professionals and between healthcare professionals and their patients.	79	88	63	88	88	86	81.5	0.550
117	To identify which remediable human factors are the most important to provide safe prescription and administration of medicines?	86	88	50	93	88	86	81.5	0.533
118	To measure which is the most sensitive moment, weekday and time of the day for occurrence of medication errors: ordering, prescribing, preparing, dispensing, administering or monitoring?	94	83	67	83	83	78	81.5	0.633
119	To investigate the effects of different interventions before/during/after the hospital discharge to ensure patients know and understand the information on their medication	94	83	56	83	83	89	81.5	0.617
120	To perform a study on nurses administering medicines in long-term care settings, what is the prevalence and root cause of medication administration adverse events?	93	88	50	88	88	83	81.4	0.550
121	To identify the impact of the availability of the Children`s Medicines List on the unmet needs of Children`s Medicines in Sub-Saharan Africa.	86	81	71	86	79	86	81.4	0.483

122	To raise awareness amongst Ministries of Health and healthcare leaders in developing countries to highlight the importance of medication safety in primary care.	88	83	61	89	83	83	81.3	0.650
123	To identify through pilot studies best practices on how to make health care facilities and health care providers accept the existing essential drug lists; and accept the use of Standard Treatment Guidelines in countries where they exist and create them where they do not exist to ensure safe, rational use of medicines.	86	81	79	81	75	86	81.3	0.550
124	To study the feasibility, impact and scalability of decision support tools to assist clinicians in safe medication prescription practice	94	81	75	75	88	75	81.3	0.600
125	To conduct a pilot intervention to develop a sharing platform among hospitals for describing errors and solutions.	88	78	83	83	78	78	81.3	0.617
126	To conduct exploratory research expanding the role of nurses to take on leadership roles in preventing medication-related patient harm.	94	88	56	88	88	75	81.3	0.583
127	What is the impact of current prescribing and medication use patterns during pregnancy on adverse birth outcomes in LMIC?	88	83	71	89	69	88	81.2	0.650
128	To create guidelines for safer use of paracetamol (acetaminophen) in children to address safe prescribing, safe and clear dosing schedule and safe dispensing and administration.	100	89	43	89	72	94	81.1	0.683
129	To conduct exploratory research into how potential medication safety issues can be predicted in a timely manner, with the use of algorithms based on clinical data.	79	79	100	79	79	71	81.0	0.517

130	To conduct a multisite study evaluating interventions that have been successful in optimising medication error reporting and learning systems.	94	81	71	72	83	83	80.9	0.567
131	To identify measures which would enable safer medication-taking practices at home in LMIC.	90	85	75	80	75	80	80.8	0.700
132	To perform a mixed method study examining the role of pharmacists and non-physicians in enhancing medication safety.	93	86	57	81	81	86	80.7	0.517
133	To investigate best practices to prevent adverse drug reactions for people with previous medication-related allergic reactions and hypersensitivities.	88	83	67	83	88	75	80.6	0.633
134	To assess how to design the best environment for learning from medication-related incidents	81	81	88	72	78	83	80.6	0.550
135	To commence a policy research on improving the impact of post-market surveillance systems to optimize patient care, drug effectiveness, and market corrections (drug recalls, etc.), including an assessment how the post-market surveillance systems differ across all countries and how this affects to medication safety.	83	83	67	79	86	86	80.6	0.433
136	To conduct an observational study in hospitals that systematically conducts medication reconciliation at discharge. How many adverse medication-related events, re-hospitalizations and deaths are avoided?	100	85	60	80	80	78	80.5	0.733
137	To identify which human factors are either involved in or contribute to medication-related patient harm	88	83	61	83	83	83	80.3	0.650

138	To identify indicators of medication safety that have been utilised in low-resource settings. What is known about their validity, reliability, and feasibility, and what potential indicators should be introduced?	88	78	67	83	83	83	80.3	0.583
139	To evaluate the economic impact of interventions targeted to reducing medication-related adverse events.	88	83	69	78	83	81	80.3	0.617
140	To design technology assisted medication administration to reduce medication errors e.g. two-tier level identification.	83	78	72	83	83	81	80.2	0.617
141	To develop and evaluate a pilot patient medication recording system based on a cloud platform, accessible to all health care providers in prescribing and reviewing a patient's medication.	81	89	83	72	89	67	80.2	0.633
142	To establish the scale and burden of inappropriate non-evidence-based use of antipsychotic and antidepressants.	81	88	81	81	81	69	80.2	0.533
143	To investigate how to increase public reporting of health care associated infections and increase rewarding/giving incentives to healthcare facilities which meet quality and safety standards	92	79	64	93	79	75	80.2	0.450
144	To identify and develop globally applicable pictograms for selected high-risk medications which would convey the critically important safety information	86	81	64	81	81	86	79.9	0.500
145	To evaluate the effectiveness of the Medication Without Harm Challenge by using Interrupted Time series to assess the implemented policies to prevent medication errors by measuring the situation prior and after (e.g. 6 months before and after) the	78	83	63	89	83	83	79.9	0.583

	introduction of the implementation of the policy								
146	To conduct a multisite workplace analysis to determine required staffing levels to achieve minimal medication errors.	93	86	64	86	79	71	79.8	0.467
147	To identify through systematic literature review best medication practices which could be implemented (modified to country context) in other countries?	94	83	67	78	78	78	79.6	0.633
148	To investigate the reliability of medication safety indicators and tools in integrated care settings.	88	81	64	81	81	81	79.5	0.567
149	To assess the influence of social determinants of health on adherence to medicines among patients with chronic diseases.	95	72	44	100	75	90	79.4	0.750
150	To identify the challenges involving the patient and their caregivers in the campaign to improve medication safety, and what resources and support is required?	94	81	81	69	75	75	79.2	0.550
151	To investigate which quality improvement factors also reduce medication-related harm.	75	83	67	75	75	100	79.2	0.450
152	To conduct epidemiology studies of adverse drug events and medical errors in non-academic tertiary settings such as nursing homes, cancer centres, and community hospitals.	94	81	69	75	75	81	79.2	0.567
153	To identify the reliable easily measured indicators to assess medication safety both at facility level and also at national level	86	81	64	88	81	75	79.2	0.483
154	To compare the efficacy of generic medication to the original	100	81	44	88	81	81	79.2	0.550

	index drug and all other generic forms?								
155	To evaluate the use of mobile technology for patients in supporting safer medicines managements, including monitoring of health conditions, education and prompting adherence?	88	83	81	83	83	56	79.2	0.550
156	To evaluate which tools can be most useful to measure medication safety and determine the impact of measures that are implemented over time	81	83	75	78	78	78	78.8	0.567
157	To assess the prevalence of drug-drug interactions where one of the interacting medicines is prescription medicine and the other is non-prescription medicine.	89	78	79	83	75	69	78.7	0.550
158	To test the use of a patient decision support system in order to ensure patient safety within self-management of medications in chronic conditions, with the remote support of health professionals and group of peers (patient experts)	81	81	63	88	81	79	78.7	0.483
159	To perform a study evaluating the strategies to improve patient monitoring of adverse effects of medication.	83	89	56	83	83	78	78.7	0.583
160	To assess through a pilot study if each time patient interacts with health care system the health care professionals would be obligated to look at least the past three visits back would reduce medication-related harm and help health care professionals to recognize medication-related harm	83	78	89	72	78	72	78.7	0.633

161	To perform a qualitative study on perceptions of community pharmacists towards the evaluation of knowledge and skills related to safe medication practice.	100	78	50	83	78	83	78.7	0.650
162	To assess the effectiveness of implementing a tool in detecting medication errors and harm in primary care?	78	89	44	89	89	83	78.6	0.633
163	To conduct a pilot study on elderly patients in a long-term care facility/nursing home, do routine medication reviews reduce the overuse of medications?	86	86	50	86	86	79	78.6	0.450
164	To perform a mixed-methods study examining the role patients and families play in contributing to medication errors due to non-adherence or over-adherence, and how much of this is due to issues with regards to access or health literacy? What evidence-based interventions exist?	85	89	69	90	70	69	78.6	0.683
165	To assess the effectiveness of annual meetings with community pharmacist, to discuss which medicines (incl. OTC medications) the patient is taking and how and when they are taking them, can reduce medication-related harm among patients who take multiple medications.	92	75	75	79	71	79	78.5	0.367
166	To identify globally applicable list of questions patients should know about their medications.	90	78	63	70	80	90	78.4	0.600
167	To investigate what are the most effective and cost-effective interventions carried out at the individual and community-level, to help reduce inappropriate antimicrobial use and reduce antimicrobial resistance.	81	78	67	83	78	83	78.4	0.633

168	To assess the health outcomes in patients with chronic conditions who receive deliberate sensitization about possible side effects, adverse effects, interactions during their visits to the clinic against another group which receives the normal (no intervention) counselling.	83	83	69	78	83	72	78.1	0.633
169	To design a patient pathway based monitoring framework and supporting metrics for evaluation of safe prescribing and administration of medicines.	67	75	83	69	100	75	78.1	0.467
170	To investigate the problem of fake medicines and their international trade from surveillance, prevention, and interdiction standpoint to get a better and more resolute idea of the true scope and prevalence of substandard and falsified medicines.	56	88	75	88	88	75	78.1	0.517
171	To develop standard protocols for the preparation, administration, monitoring of insulin in acute care settings, for application internationally.	94	75	50	81	75	94	78.1	0.550
172	To identify best strategies to avoid inappropriate self-medication with antibiotics	88	88	44	88	75	88	78.1	0.567
173	To perform a cross-sectional study across primary care facilities assessing medication safety and exploring strategies in which it can be improved.	81	81	56	88	81	81	78.1	0.567
174	To identify through literature review most effective interventions for medication safety and evaluate and measure their effectiveness through piloting sites using pre-set indicators.	94	81	63	81	75	75	78.1	0.533

175	To increase awareness among regulators and pharmaceutical industry how look-alike containers, poor visibility of key information (e.g. Active pharmaceutical ingredient) can cause medication errors and assess if dispensing only the necessary dose (breaking the fixed package when needed) would increase rational use of medicines.	71	79	75	79	79	86	78.0	0.400
176	To assess the role of regulators, manufacturers and the pharmaceutical industry in reducing medication related harm.	75	86	64	86	79	79	78.0	0.467
177	To create a cost-effectiveness study to identify mechanisms that support safe prescribing for elderly, poly-medicated patients.	79	75	69	75	88	81	77.7	0.467
178	To develop clinical guidelines for rarely used drugs and perform audits on use.	94	69	69	88	69	79	77.7	0.500
179	To test through pilot sites which are most fruitful and powerful combinations of practices, processes, and tools to prevent avoidable medication-related harm.	78	78	75	83	78	72	77.3	0.633
180	To form and divulge basic notions about antibiotics, their uses, operations and sequential effects for patients to understand in what moments it is correct to prescribe them and the importance of following the guidelines recommended by their physician.	90	75	50	85	80	83	77.2	0.633
181	To assess the effectiveness of different implementation strategies for sustaining and/or scaling up evidence-based interventions to reduce, eliminate or ameliorate medication-related patient harm?	81	75	81	75	75	75	77.1	0.467

182	To conduct and evaluate the impact of medication reconciliation practices during transitions of care (e.g. post-hospital discharge).	85	85	60	80	80	72	77.0	0.700
183	To develop and evaluate a system that monitors for the incompatibility of chemotherapy drugs with medications prescribed for other comorbidities, to reduce unwanted synergies.	83	86	58	83	79	71	76.8	0.400
184	To perform an observational study to identify which laboratory tests can early diagnose a medication error.	75	67	100	64	71	83	76.8	0.467
185	To identify and evaluate the primary national systems and/or strategies in place for identifying the burden and scope of medication-related harm in high-, middle-, and low-income countries?	79	81	50	81	81	88	76.6	0.550
186	To identify the role of different actors in health care in reducing global incidence of medication related adverse events.	78	89	50	83	78	81	76.5	0.600
187	To investigate how technologies could be appropriately implemented and scaled in LMICs to better ensure that drugs are not spoiled, diverted, counterfeited, and that supply chain performance is optimized to avoid stock outs and drug shortages.	71	81	63	81	81	81	76.5	0.500
188	To investigate the impact, feasibility and scalability of patient education techniques to promote reconciliation and adherence delivered in a variety of ways including: pharmacy, clinic, telephone, smart phone application.	89	80	75	75	80	60	76.5	0.650

189	To perform a cost-effective analysis on interventions to reduce preventable medication-related hospitalizations , and medication-related falls and injuries in older adults.	83	88	50	83	72	81	76.3	0.600
190	To identify weak points in the design of medication and/or its packaging and develop changes which will help reduce mistakes in medication administration.	79	79	58	81	75	86	76.2	0.483
191	To investigate what percentage of medication errors are due to similarities in medication appearance.	89	75	67	85	75	67	76.2	0.600
192	To assess the medication safety practices in long-term care facilities	100	79	43	79	79	79	76.2	0.517
193	To conduct a study examining the role of individual health professionals and inter-professional collaboration, who is accountable for ensuring medication safety?	79	75	69	81	75	79	76.2	0.467
194	To investigate the role of pharmacist's intervention in reducing medication errors through prospective cohort study. In which ways can the role of pharmacists be expanded to be actively involved in the medication prescribing process.	80	88	56	83	78	72	76.2	0.550
195	To identify the challenges and opportunities for health care professionals and the health care system to improve medication safety in transitions of care.	81	78	56	83	78	81	76.2	0.567
196	To design a uniform global standard of labelling for OTC medications and create recommendations for good labelling practice, including use of simple language.	88	81	56	78	67	88	76.2	0.500

197	How to adapt patient safety indicators obtained from routinely collected data to different contexts and countries worldwide?	69	81	69	75	81	81	76.0	0.467
198	To identify gaps in deprescribing potentially inappropriate medicines in older people.	88	81	50	81	81	75	76.0	0.467
199	To conduct intervention study which utilizes mixed methods approach to transform a unit/hospital with high prevalence of medication errors to a low prevalence.	69	81	63	81	88	75	76.0	0.483
200	To investigate the prevalence of adverse drug events and medication errors contributed by extemporaneous compounding medications for children, due to limited access to safe and effective medicines.	75	80	56	80	80	85	75.9	0.700
201	To conduct a qualitative study seeking to examine how to enhance patient engagement amongst patients and their family, to improve medication safety.	89	72	67	78	78	72	75.9	0.583
202	To create up-to-date recommendations for safe and good prescribing practices.	94	78	50	83	78	72	75.9	0.567
203	To identify methods of monitoring patient medication adherence and develop evidence-based practices to improve adherence, reduce patient harm and improve outcomes.	100	83	44	83	78	67	75.8	0.600
204	To assess how innovative models of pharmaceutical care and diffusion of technology can be used to reduce medication related harm.	72	81	57	78	78	88	75.6	0.483

205	To assess how in resource poor countries/regions, evidence-based medication management systems which cover from prescribing to administering drugs can be implemented, and what organizational and cultural requirements are needed to implement such systems?	71	79	79	69	75	81	75.6	0.500
206	To conduct a multi-professional study implementing different interventions to investigate the effect on reducing medication errors.	75	79	64	79	79	79	75.6	0.383
207	To conduct a study on enhancing the role of pharmacists in multidisciplinary collaboration with prescribers. What are the attitudes of pharmacists and prescribers, how will collaboration best be utilised?	88	79	50	81	75	81	75.6	0.500
208	To perform a study on the role of pharmacists in elderly patients transitions of care. What is their role, how can it be improved and what is the impact?	81	80	55	80	85	72	75.6	0.600
209	To critically evaluate procedures in the transfer of medication process to ensure safe inter-unit transfer.	83	78	61	78	78	75	75.5	0.583
210	To examine each step of the medication process, what is the impact of interruptions in different stages and what implements can be made for improvement?	72	78	75	78	78	72	75.5	0.600
211	To conduct a study investigating the impact of procurement based on clinical efficacy and safety, with the use of longitudinal data analytics thereby optimising benefits and minimising harm.	80	80	75	67	80	70	75.3	0.317

212	To develop and validate models focused on aspects of hospital layout and healthcare worker/patient flow to reduce HAIs	80	75	63	83	80	70	75.1	0.300
213	How does operationalization of 'resilience' in the health care system affect medication safety?	75	67	80	67	75	88	75.1	0.333
214	To evaluate forms of optimizing drug prescribing in the elderly in collaboration of pharmacists and GPs.	78	78	50	78	89	78	75.0	0.567
215	To assess cost-effective ways to ensure medication compliance and investigate the role of mobile technology/assistive technology, to increase compliance in resource poor settings.	88	75	75	88	75	50	75.0	0.583
216	To assess the human factors which affect the decision-making process during prescribing, to teach best practices to doctors.	89	78	56	83	72	72	75.0	0.633
217	To examine ambulatory practice prescribing, which types of medications, or combinations of medications, have a high risk of safety problems in patients.	75	78	63	83	83	67	74.8	0.633
218	To identify pictograms which could be used to teach patients about their medicines	86	81	50	81	75	75	74.7	0.467
219	To identify strategies to improve compliance of medication in patients, caregivers and health professionals. What are effective newly developed tools for medication safety?	88	75	58	81	75	69	74.3	0.533
220	To reduce harm related to interactions by supporting creation of an app which would help prescribers in reducing the risk of interactions, supporting in decision making, offering possible alternative options, such as	88	78	75	78	72	56	74.3	0.533

	deprescribing, while considering patient's pathologies.								
221	To create a comparative study, which would assess the effectiveness of standardized, patient carried basic medication list in a developing country compared to more involved and complex tools (e.g. Best possible medication history lists) used in developed countries	75	70	70	80	70	80	74.2	0.600
222	To develop a mobile technology tool for patient and clinician use, aiding appropriate polypharmacy in LMIC.	83	83	61	72	83	61	74.1	0.533
223	To investigate the response to opioids in middle-income countries: is the rise of opioid misuse, abuse, dependence associated with the increased availability of extended-release opioids.	81	63	69	81	63	88	74.0	0.517
224	To perform a qualitative study within the health care workforce focusing on what style of leader health care professionals desire to prevent avoidable medication-related harm?	71	69	88	75	69	71	73.8	0.533
225	To conduct a study amongst health practitioners identifying at the practitioner-level barriers to implementation of evidence-based health literacy practices for explaining medications to patients and their families.	69	75	81	61	72	83	73.6	0.533
226	To investigate the impact of changing the package on the uptake of safe medication practices.	88	71	67	69	69	79	73.6	0.467

227	To implement an information campaign which would aim to inform population in in a more conscientious way about traditional medicines and food supplements possible risks and interactions with their usual medication.	89	69	63	72	61	88	73.5	0.517
228	To create effective techniques in medication error disclosure between health care professionals and to the patient, including legal statutes that can promote transparency.	71	79	67	79	79	67	73.4	0.367
229	What strategies can be implemented to reduce the overuse of medications with poor effectiveness in terminally ill cancer patients?	69	75	63	88	75	71	73.4	0.483
230	To assess the risk of polypharmacy for individual patients and develop cost-effective solutions.	81	72	61	72	78	75	73.3	0.567
231	To assess the clinical significance of drug interactions based on recent studies and creating evidence-based strategies in deprescribing.	81	78	56	78	72	75	73.3	0.600
232	To gather evidence about the clinically important role of pharmacists in medication safety.	81	81	44	78	78	78	73.3	0.500
233	To conduct a study investigating patient stock-piling of medications and conditions of their storage, their beliefs related to use of medicines and sources of medication information.	100	67	61	72	72	67	73.1	0.583
234	To determine the incidence of antibiotics obtained without prescription or from earlier consultation by a physician, and how information technologies can reduce risky behaviours in LMIC.	72	78	67	72	72	78	73.1	0.533

235	To investigate the prescribing indication for opioids in middle- or third-world countries, has liberal prescribing for end-of-life care transitioned into use for non-cancer pain management.	88	63	69	75	63	81	72.9	0.483
236	To create an evaluation framework that measures improvement initiatives so that those can be compared (by how effective, efficient, timely, patient-centred, equitable and safe each intervention is).	81	69	69	75	75	69	72.9	0.433
237	To conduct a longitudinal study on how often systematic medication reviews should be performed in nursing home residents and how medication prescribing should be adjusted as treatment goals shift form preventive/curative to palliative/comforting care.	92	67	67	75	67	70	72.8	0.333
238	To address the key issues in reconciling prescriptions and administration across settings through a pilot study.	100	72	56	67	72	69	72.6	0.617
239	To assess various human factors, such as level of informance on medications, as variates to measure which human factors in patients and health care professionals effect prevalence of medication errors.	79	79	42	79	79	79	72.4	0.400
240	To investigate the change in medication safety practice after presenting medication error root cause analysis to healthcare professionals.	79	78	50	78	75	75	72.4	0.500
241	To assess nurses' skills to do dose calculations after absence from work and compare the drug calculation skills of recent nursing students to experienced nurses who have just came back to work	86	67	90	83	58	50	72.3	0.367

	from leave which length has been predetermined to be significant enough (e.g. in maternity leave)								
242	To critically evaluate implementation of a standardized opioid tolerance assessment and documentation prior to administration.	100	67	58	64	64	79	72.0	0.400
243	To assess how different interventions (including monitoring systems) reduce incidence of harm due to drug-drug interaction in poly-medicated elderly patients.	81	78	56	72	72	72	71.9	0.500
244	To identify strategies to increase adverse drug event reporting/post marketing surveillance amongst physicians.	90	90	45	80	65	61	71.9	0.650
245	To assess the role of nurses in preventing drug-drug interactions.	89	67	67	72	61	75	71.8	0.517
246	To explore novel ways, we can utilise technology (applications and other automation) to reduce adverse medication-related events. What is the role for technology in assisting patient monitoring and correct use of medications?	75	78	67	78	78	56	71.8	0.550
247	To design and evaluate effective interventions designed to improve the uptake of health literacy practices related to medication teaching.	71	79	50	79	86	64	71.4	0.367
248	To implement national level controlling and monitoring of safety of herbal medications.	86	71	50	81	69	71	71.4	0.433
249	To identify the predictor factors for patient readmission to hospital for medication-related problems in patients with complex conditions.	88	72	69	72	67	61	71.4	0.567

250	To investigate the relationship between the increased availability of prescription opioids and increased illicit drug use.	81	69	71	69	63	75	71.3	0.433
251	To identify and build a platform which would allow patients to access their up-to-date medication list in all times regardless of the physical location.	75	75	75	65	75	61	71.0	0.617
252	To quantify the impact of electronic systems and knowledge-based clinical decision support on reducing medication-related harm in LMICs, by implementing this technology to smart-phones.	69	72	78	72	72	61	70.7	0.483
253	To conduct an exploratory study on the conditions and regulations needed to adopt the prescription to OTC switch.	75	70	75	67	50	88	70.7	0.333
254	Can implementing interventions related to barriers to de-prescribing, in patients with multi-morbidity and polypharmacy, minimise morbidity, mortality, reduce financial burden, improve medication adherence leading to enhance patient safety?	75	71	58	71	64	83	70.6	0.383
255	To conduct an observational study examining the handling of high-risk medications across the continuum of care, and to identify strategies to ensure its safe use.	89	78	50	75	70	61	70.5	0.567
256	To conduct an experimental study investigating the differences in the length of carriage of resistant bacteria, after exposure to a single course of antibiotics.	75	69	79	61	67	72	70.4	0.483
257	To assess patient attitudes and behaviours towards use and storage of medications in relation to health outcomes.	94	67	56	78	67	61	70.4	0.500

258	To assess through an observational study how adverse drug events happen and implement interventions to prevent them from occurring.	70	80	50	75	80	67	70.3	0.600
259	To conduct an intervention study on the impact of medication dispensing technology in preventing and/or reducing medication errors.	69	72	61	72	72	75	70.3	0.467
260	To assess the consequences to the individual's well-being and to their effectiveness when the workplace pursues complete elimination of avoidable harm.	58	67	83	75	67	70	70.0	0.350
261	To perform a study targeting health care prescribers, addressing how to promote effective communication and collaboration amongst other prescribing providers.	75	75	50	75	75	70	70.0	0.283
262	To identify the best practices for outpatient medication reconciliation followed by creating a toolkit out of the best practices (incl. interventions aimed at patients/caregivers, staff, clinics, health systems, and communities)	83	72	50	72	72	69	69.8	0.467
263	To perform a cost-effective analysis on interventions to improve medication adherence to evidence-based cardiovascular pharmacotherapy and other chronic conditions.	75	78	56	83	63	64	69.7	0.517
264	To identify which factors contribute to administration errors where medication is not given in nursing homes and care homes	75	75	60	75	67	67	69.7	0.300
265	To conduct a qualitative study for patients, what are their priorities for reducing the risk of medication-related harm?	85	61	75	75	55	65	69.4	0.550

266	To assess practices which can increase or decrease errors in anticoagulant therapy of patients who have transitions of care between secondary and primary care.	75	75	60	75	80	50	69.2	0.350
267	To evaluate incidents of inappropriate polypharmacy and develop strategies to reduce harm and improve patient health outcomes.	81	72	50	72	72	67	69.1	0.467
268	To research into the utilization of digital health services monitoring large population health records to survey adverse drug reaction signals.	57	63	81	69	81	63	68.9	0.433
269	To assess the benefits technology can play in reducing medication errors caused by verbal orders, during transition of care and in engaging and educating patients on their medication.	64	78	63	72	78	56	68.5	0.483
270	To identify factors that influence patient decision-making about polypharmacy, to develop decision aids for patients to help them take part on the discussion of taking multiple medications.	81	67	50	72	72	67	68.2	0.467
271	To conduct a study into patient-user strategies, such as e-health and patient information, as facilitators for safe medication use and medication adherence.	71	75	75	69	69	50	68.2	0.450
272	To analyze and identify the root cause of multidrug resistance in the treatment process to create more effective interventions.	81	81	44	67	72	63	67.9	0.517
273	To conduct a study within long-term care/nursing home facilities, examining the surveillance and reporting systems of adverse drug events and patient harm due to atypical antipsychotics.	79	57	60	83	64	64	67.9	0.333

274	To investigate patient access and use of online medication record systems (such as OpenNotes) and medication schedules.	86	71	79	57	71	43	67.9	0.400
275	To perform an intervention study evaluating whether the provision on information about patient therapy to community pharmacists leads to a decrease in medication errors.	79	71	50	57	71	75	67.3	0.367
276	To conduct exploratory research on emerging technologies to prevent medication events from occurring, available for use in the patient's home and/or long-term care settings.	61	72	69	72	67	61	67.0	0.467
277	To conduct a multisite study analyzing the transfer and communication of medication-related information across health care settings, and between patients and providers. What are the weak points, how can current practices be strengthened?	63	64	75	69	63	69	67.0	0.417
278	To systematically evaluate the most appropriate devices to deliver intravenous medications assessing the risk of complications, and to additionally investigate.	75	63	63	86	64	50	66.7	0.467
279	To create a study comparing effectiveness of two systems, one where medication errors and adverse drug reactions are reported to different entity/portal, and second where these two are combined and the approach is integrated.	93	57	64	57	64	64	66.7	0.367
280	To determine factors that drive spread of HAIs and investigate new approaches that minimize the role of the healthcare environment in the spread of germs	80	60	50	80	60	70	66.7	0.267

281	To apply global standards on packaging and labelling to have uniformed place and way how and where the best before date and strength of the medicine is placed in the product.	83	67	42	67	67	75	66.7	0.367
282	To investigate the impact of personalised medicines.	58	67	75	58	67	75	66.7	0.333
283	To implement data analytics to reduce medication related patient harm.	83	75	42	75	67	58	66.7	0.317
284	To conduct research into the development of expert systems encompassing a wide scope of patient information (including age, gender, genetic makeup, laboratory tests), to aid as a clinical decision support.	67	67	67	67	67	67	66.7	0.333
285	To conduct an intervention study in hospital emergency departments evaluating the effectiveness of strategies (organizational/functional) to reduce delay and overcrowding.	81	64	36	79	57	79	65.9	0.417
286	To review current prescribing guidelines and develop strategies to reduce overprescribing, particularly for opioids.	83	78	39	67	67	61	65.7	0.533
287	To conduct a cost-effective analysis on interventions to reduce avoidable drug-related hospital admissions.	83	72	50	67	61	61	65.7	0.517
288	To design and develop sustainable, childproof and tamperproof containers which would ensure there is no risk to take dose higher than intended (e.g. Impossible to dispense more than 10 ml before realigning it)	75	63	56	56	63	81	65.6	0.417
289	To perform a study identifying medications (and/or combination of medications), that can potentially lead to acute kidney	93	63	43	69	63	63	65.3	0.433

	injury in high-risk critically ill patients.								
290	To investigate incidents of medication-related patient harm, what role and to what extent does technology and technical complexity contribute?	64	69	71	69	75	44	65.3	0.417
291	To conduct a study investigating clinical situations that lie outside the guidelines, is there an increased incidence of unnecessary bridging with heparin or low molecular weight heparin?	90	67	60	67	50	58	65.3	0.283
292	To create a study assessing long-term adherence and compliance of patients who have complex multi-medication management, by looking from both the perspective of the patient and health care professional.	85	70	28	80	65	60	64.6	0.617
293	To identify and examine different types of surveillance programmes utilised to monitor medications with the potential for abuse.	88	56	63	63	63	56	64.6	0.400
294	To examine whether the involvement of Health IT is functioning as expected to improve medication safety.	75	69	50	75	69	50	64.6	0.400
295	To trial a pilot intervention study of the barcode system for medication in LMIC.	81	67	67	61	56	56	64.5	0.467
296	To assess improvements in medication safety after introducing restrictions on drug dispensing (requirement for a valid prescription).	67	75	50	64	64	64	64.1	0.333
297	To examine the impact of using external, non-biased safety testing companies, in evaluating the risks associated with potential brand names.	75	67	58	67	67	50	63.9	0.317

298	To assess how decision support systems can be integrated with patient data, the culture barriers to rational prescribing in resource poor countries and how electronic prescribing systems relate to traditional medicine	56	71	69	63	64	57	63.4	0.400
299	To investigate the global burden of opioid over-doses and related deaths.	81	64	29	63	69	75	63.4	0.433
300	To evaluate the implementation of QR coding for all medicines in their respective market and assess the public awareness of the need to verify the authenticity of medicines in countries which have implemented the QR coding	63	60	88	60	60	50	63.3	0.217
301	To conduct a multisite analysis investigating hospital adverse events. What has been the impact of electronic medical record (EMR) systems, the different modes of EMR, and optimal approaches in design and delivery?	70	67	58	67	67	50	63.1	0.300
302	To conduct a study designed to investigate the intentional non-adherence of medication.	72	61	50	78	61	56	63.0	0.483
303	To compare the benefits of pictorial information in medication instructions to written instructions alone, in improving medication safety. To what extent, in what contexts and formats is pictorial information most beneficial?	69	63	56	63	63	63	62.5	0.417
304	To compare generic marking of every individual medication and dosage against existing medication in improving medication safety?	63	60	75	67	50	60	62.4	0.300
305	To research into the development of novel electronic health record data entry methods, to facilitate accuracy and reduce errors.	71	63	61	72	61	44	62.1	0.433

306	To develop Shared Care Guidelines for selected medicines, to promote safe continuity of care in the community.	86	64	36	64	64	58	62.1	0.350
307	To assess the benefits and risks of medications affecting the central nervous system, such as antipsychotics, antidepressants, anticonvulsants, benzodiazepines, in patients with complex conditions such as ADHD, substance abuse/misuse, and chronic pain.	64	72	42	63	69	63	62.0	0.400
308	To ascertain and develop the role of clinical informaticians to bridge the gap between health IT design implementation and healthcare professionals.	69	57	64	63	63	56	61.9	0.383
309	To investigate the effect on patient safety if medication is infused through central versus peripheral veins.	75	69	43	69	63	50	61.3	0.467
310	To identify through pilot studies ways to prevent inappropriate use and over-prescribing due to financial incentives to physicians.	50	69	69	50	63	63	60.4	0.367
311	To investigate and develop model success stories utilising health IT.	81	63	56	63	50	50	60.4	0.433
312	To evaluate the current electronic prescribing process and pilot a redesign on indication and decision supporting drug of choice.	67	75	67	67	67	20	60.3	0.300
313	To undertake exploratory research on the feasible implementation of a feedback system for physician prescribing practices.	56	61	72	56	56	61	60.2	0.433
314	To conduct a longitudinal observational study of patient medication non-adherence on health outcomes.	88	63	25	67	67	50	59.7	0.517

315	To conduct a study exploring implementation methods of drug classification systems in LMIC.	67	57	50	64	50	67	59.1	0.300
316	To evaluate effectiveness of treatments used to manage extravasation of intravenous medications to minimize medication related harm.	70	71	25	57	71	57	58.7	0.350
317	To conduct an intervention study to determine whether handling of intravenous fluids alike medications (with protected access and patient-specific details) will reduce medication-related harm.	58	57	58	58	58	60	58.4	0.300
318	To conduct a multisite/multimodal study evaluating the role and impact of utilising digital methods in the health care process to reduce medication errors.	50	63	57	63	63	50	57.4	0.367
319	To identify high-risk situations applicable globally that should be programmed in all information systems (EHRs, CPOE, e-prescribing).	57	57	64	57	57	50	57.1	0.317
320	To identify and create recommendations for the most effective approach to decision support alerts in electronic prescribing systems, the optimum sensitivity and specificity and criteria which should be used to enable prescriber to receive alerts but not receive alert fatigue.	67	63	50	63	63	38	56.9	0.267
321	To investigate how having an understanding about health literacy can contribute towards ways that practitioners give information to patients about their medications.	63	61	28	67	56	67	56.7	0.467

322	To study the feasibility, impact and scalability of awareness among patients and clinicians, that low kidney function as a major risk factor for medication-related patient safety events, delivered in a variety of ways - pharmacy, clinic, telephone, smart phone application?	70	58	70	58	50	33	56.7	0.283
323	To develop digital thermometers for use with medicine fridges and freezers.	78	50	39	67	50	44	54.5	0.417
324	Assessing the benefits on patient safety and efficacy of marking expiration month and date on tablets.	69	50	33	81	44	50	54.5	0.383
325	To conduct exploratory research on new methods to improve user-centred design and technology.	60	50	50	50	60	50	53.3	0.250
326	To evaluate the efficacy of generic antibiotics compared to their original patented brand. Do they have the same impact on antibiotic-resistant bacteria in the digestive flora?	50	50	50	58	50	58	52.8	0.267
327	To assess the efficacy of antibiotic delivery by the per lingual route, what is the impact on the gut flora and contribution to antimicrobial resistance?	60	50	50	43	57	50	51.7	0.300
328	To examine the role of Artificial Intelligence in reducing medication-related patient harm from the perspective of multiple stakeholders including clinicians, patients, consumers and administrators.	60	38	63	50	50	40	50.0	0.250
329	To research into producing a medicines handbook that classifies medication by disease and patient group, that can be applied to different geographic country contexts.	50	38	63	50	38	50	47.9	0.383

330	To assess the required global standards required to be in place before introducing a new digital system to any market.	63	43	43	50	44	44	47.6	0.383
331	To identify ways to ensure that the systemic problems (and failings) of medication safety amongst healthcare professionals will not conflict with the current trend of increasing patient knowledge and awareness.	44	44	63	44	38	29	43.3	0.367
332	To create recommendations to accurately identify a patient which could be applied to different institutional contexts.	42	42	30	42	25	25	34.2	0.333
333	To investigate the change in the status of the medication to create reliable processes.	25	30	20	25	25	25	25.0	0.317
